# Multiple Genetic Alterations within the PI3K Pathway Are Responsible for AKT Activation in Patients with Ovarian Carcinoma

**DOI:** 10.1371/journal.pone.0055362

**Published:** 2013-02-07

**Authors:** Carmela De Marco, Nicola Rinaldo, Paola Bruni, Carmine Malzoni, Fulvio Zullo, Fernanda Fabiani, Simona Losito, Marianna Scrima, Federica Zito Marino, Renato Franco, Alfina Quintiero, Valter Agosti, Giuseppe Viglietto

**Affiliations:** 1 Department of Experimental and Clinical Medicine, University Magna Graecia, Catanzaro, Italy; 2 Biogem Scarl, Institute for Genetic Research “Gaetano Salvatore”, Ariano Irpino, Avellino, Italy; 3 Casa di Cura “Malzoni-Villa dei Platani”, Avellino, Italy; 4 Fondazione “G Pascale”, National Cancer Institute, Naples, Italy; The University of Hong Kong, China

## Abstract

The phosphatidylinositol 3-kinase (PI3K)/AKT pathway is activated in multiple cancers including ovarian carcinoma (OC). However, the relative contribution of the single components within the PI3K pathway to AKT activation in OC is still unclear. We examined 98 tumor samples from Italian OC patients for alterations in the members of the PI3K pathway. We report that AKT is significantly hyperactive in OC compared to normal tissue (n = 93; p<0.0001) and that AKT activation is preferentially observed in the elderly (>58 years old; n = 93; p<0.05). The most frequent alteration is the overexpression of the p110α catalytic subunit of PI3K (63/93, ∼68%); less frequent alterations comprise the loss of PTEN (24/89, 27%) and the overexpression of AKT1 (18/96, 19%) or AKT2 (11/88,12.5%). Mutations in the PIK3CA or KRAS genes were detected at lower frequency (12% and 10%, respectively) whereas mutations in AKT1 or AKT2 genes were absent. Although many tumors presented a single lesion (28/93, of which 23 overexpressed PIK3CA, 1 overexpressed AKT and 4 had lost PTEN), many OC (35/93) presented multiple alterations within the PI3K pathway. Apparently, aberrant PI3K signalling was mediated by activation of the canonical downstream AKT-dependent mTOR/S6K1/4EBP1 pathway and by regulation of expression of oncogenic transcription factors that include HMGA1, JUN-B, FOS and MYC but not by AKT-independent activation of SGK3. FISH analysis indicated that gene amplification of PIK3CA, AKT1 and AKT2 (but not of PI3KR1) and the loss of PTEN are common and may account for changes in the expression of the corresponding proteins. In conclusion, our results indicate that p110α overexpression represents the most frequent alteration within the PI3K/AKT pathway in OC. However, p110α overexpression may not be sufficient to activate AKT signalling and drive ovarian tumorigenesis since many tumors overexpressing PI3K presented at least one additional alteration.

## Introduction

Epithelial ovarian carcinoma (OC) is the most lethal gynecological malignancy with most patients diagnosed only at advanced stages [1], [2]. OC is a complex disease that exhibits remarkable heterogeneity at the clinical, cellular and molecular level. [3]–[6] OC is classified by histotype and grade. Histologically, OC is classified into serous (S-OC), mucinous (M-OC), endometrioid (E-OC), clear cell (CC-OC), transitional (or Brenner cancer), squamous cell, and undifferentiated types. S-OC are the most common type of OC, accounting for about two thirds of ovarian carcinomas [7]. As to the grade, Type I lesions constitute 10–20% of OC and include low-grade S-OC, M-OC E-OC, and CC-OC. Low-grade type I cancers present in early stage (I–II), show low-malignant potential, grow slowly, and are relatively resistant to platinum-based chemotherapy. Conversely, Type II lesions include high-grade S-OC, and undifferentiated cancers that present at late stage (III–IV), grow more aggressively, though they respond more frequently to platinum-based treatment. The cure rate of patients affected by OC remains low (approximately 30%) although the outcome of patients in the late stage has recently improved, with 5-year survival rates approaching 50% [8]–[10].

OC represent independent diseases, being characterized by genetic changes that are remarkably different in Type I and Type II OC. Type I low-grade OC are almost euploid, retain wild-type p53, and are apparently driven by activating mutations of *RAS* and *PIK3CA*, and inactivating mutations of *PTEN*. Conversely, type II high-grade OC almost invariably present genomic instability caused by mutation and/or silencing of *BRCA1* or *BRCA2* and *p53* mutation [11]. Such genomic instability results in diverse subsequent events that include alterations within the PI3K/AKT pathway, which are believed to drive tumor growth and metastatic progression, [3], [12]–[14].

The PI3K/AKT pathway is activated in multiple cancers leading to oncogenic transformation [15], [16]. The mechanisms responsible for activation of the PI3K/AKT pathway in human cancers are diverse and include dysregulation of growth factor receptor and integrin signalling, activating RAS mutations, activating mutations or gene amplification of the gene encoding the p110α catalytic subunit of PI3K (PI3KCA), inactivating mutations in the phosphatase and tensin homolog (PTEN) tumor suppressor gene or in the gene encoding the p85 regulatory subunit of PI3K (PI3KRA).

Previous studies have shown aberrant activation of PI3K/AKT pathway in OC. Reportedly, OC show phosphorylation of different AKT isoforms [3], [12], [13]. AKT activation is common in high-grade, late-stage serous OC [17]–[20] and may therefore play a role in mediating tumour progression. In addition, PIK3CA and AKT2 genes are amplified in OC and gain-of-function mutations have been detected in PIK3CA and AKT1 [21]–[25].

Recently, a multiplatform genomic analysis by The Cancer Genome Atlas (TCGA) Research Network identified alterations in the PI3K/AKT and RAS pathways in approximately 45% of high-grade S-OC [11]. Here, we performed an integrated analysis of OC in an Italian cohort of patients in order to characterize the molecular mechanisms that lead to the activation of the PI3K/AKT pathway in OC.

## Materials and Methods

### Ethics Statement

Patient accrual was conducted according to Institutional Review Board of the AOU Mater Domini/University Magna Graecia (Catanzaro, Italy) and Casa di Cura “Malzoni-Villa dei Platani” (Avellino, Italy). The study was approved by the Institutional Review Board of the AOU Mater Domini/University Magna Graecia in the meeting of October 28^th^ 2011. Written informed consent was obtained from all participants to the study.

### Patients

Archive material from 98 patients diagnosed of OC was obtained from the gynaecological Units of Casa di Cura “Malzoni-Villa dei Platani” (Avellino, Italy) and University Magna Graecia. Patients were surgically staged according to FIGO (International Federation of Gynaecology and Obstetrics) criteria (Cancer Committee of the International Federation of Gynaecology and Obstetrics, 1986). Patient diagnosis was made according to the WHO (World Health Organization) criteria [26]. Median age was 58 year old (range 21–86). See Table S1 for more detailed clinical characteristics of patients.

### Tissue Microarray (TMA) and Immunohistochemistry

TMAs (257.1 and 257.2) were constructed in collaboration with the Unit of Immunostaining at the Centro Nacional de Investigaciones Oncologicas (Madrid, Spain) according to established methods [27] using a Tissue Arrayer (Beecher Instruments, Gene Micro-Array Technologies, Silver Spring, MD). Two cores of ovarian carcinoma were arrayed from each case. TMA slides were deparaffinized, heated in a pressure cooker with 1 mM EDTA, pH 8.0 for 10 min, and incubated with pepsin at 37°C for 30 min. Slides were then dehydrated in increasing ethanol concentrations, and then air-dried. Probes were denatured at 96°C for 5 min, and hybridization solution was applied on each slide and incubated at 75°C for 1 min. After overnight incubation at 37°C in a humid chamber, slides were washed with 0.4 X SSC and 0.3% NP40 for 2 min at 75°C, air-dried in darkness, counterstained with DAPI, and a coverslip was applied.

Immunostaining was performed using the avidin-biotin-peroxidase method (LSAB kit; DAKO, Glostrup, Denmark) as described previously [28]. Antibodies used for immunostaining were selected according to previously published works [29]–[36]. Anti-phospho Akt (S473) (#9277), anti-AKT1 (#2938), AKT2 (#4057), PIK3CA (#4249), PTEN (#9559), anti-phospho-mTOR (Ser2448) (#2971), anti-phospho-p70 S6 kinase (Thr389) (#9206), anti-phospho-4EBP1 (Thr37/46) (#2855), anti-phospho-S6 (Ser235/236) (#2211), anti-phospho SGK3 (Thr320) (#5642) were all from Cell Signaling Technology (Danvers, MA, USA); anti-PIK3R1 (#610046) was from BD Transduction Laboratories.

The immunohistochemical score of pAKT and pSGK3 used in this work was selected on the basis of widely established criteria existing in the literature [31] by multiplying the percentage of labelled cells (ranging from 0% to 100%) by the intensity of the staining (1-weak, 2-moderate and 3-strong). Scores above 150 were considered positive (+).

For the immunostaining scores of AKT1, AKT2, PIK3CA, PIK3R1 and PTEN, we selected criteria described in previous studies [32], [33], [37]–[39]. The immunoreactivity was evaluated considering both the percentage of positive cells (score: 0–3 for respectively, <5, 5–25, 25–50, >50%) and the intensity (score: 1–3) of staining. The product of both yield a final immunostaining score: 0, −; 1–3, +; 4–6, ++; and 7–9, +++). For statistical analysis, tumours were classified into a low expression group comprising (−) and (+), a moderate expression group comprising (++) and a high expression group that comprises (+++). PTEN expression was considered lost (−) when scored 0–3, reduced (−/+) if the score was 4–6 and positive (+) for 7–9 score.

The staining of phospho-mTOR, phospho-p70 S6 kinase, phospho-4EBP1, phospho-S6 was scored as described before [40].

### Western Blot and Antibodies

Whole cell extracts were homogenized in NP-40 lysis buffer (10 mM Tris–HCl pH 7.5, 150 mM NaCl, 1% NP-40) containing protease and phosphatase inhibitors (Sigma-Aldrich). After incubation on ice for 30 min, samples were centrifuged at 12000 rpm at 4°C for 30 min. Equal amounts of proteins were separated by 6–15% SDS-PAGE and transferred to nitrocellulose membrane (Whatman). Membranes were incubated overnight at 4°C with the following antibodies: anti-PIK3CA (#4249), anti-phospho-Akt (Ser473) (#4058), anti-Akt (#9272), anti-phospho-mTOR (Ser2448) (#2971), anti-phospho-p70 S6 kinase (Thr389) (#9206), anti-phospho-4EBP1 (Thr37/46) (#2855), anti-phospho-S6 (Ser235/236) (#2211) were purchased from Cell Signaling Technology (Danver, MA); anti-β-Actin (clone AC-74, #A2228) was from Sigma-Aldrich.

### Fluorescence In Situ Hybridization (FISH)

FISH analysis was performed on TMAs. BAC clones were designed according to the Ensembl database (www.ensembl.org). BAC clones covering the AKT1 gene were RP11-982M15, RP11-477I4 and RP11-556J09. Control BAC probes covering chromosome region 14q11 was RP11-324B11. BAC clones covering the AKT2 gene were RP11-36B02, RP11-688J23, RP11-725P04. Control BAC probes covering chromosome region 19p13.1 were RP11-737I1, RP11-520G3. BAC clones covering the PIK3CA gene were RP11-360P21 and RP11-245C23. Control BAC probes covering chromosome region 3p14.1 were RP11-175F9 and RP11-15B21. All BAC clones were labelled with dUTP-Sprectrum Orange (Vysis Inc., DownersGrove, IL; USA). All Control probes were labelled with dUTP-Sprectrum Green (Vysis Inc., DownersGrove, IL; USA).

Two different investigators (R.F., S.L.) that had no previous knowledge of the genetic, clinical and IHC results evaluated FISH analysis. All FISH were scored in an average of 130 (60–210) nuclei. For evaluation of copy number of the genes encoding AKT1, AKT2 and PIK3CA, a gene-to-control ratio of 1.0 was classified as disomy; ratios between 1.0 and 2.0 were considered gene low-level gains; ratios >2.0 were considered as high polysomy and/or gene amplification [41], [42]. Accordingly, tumours were divided into different classes: disomy, trisomy (3 copies of chromosomes in >40% of cells), low polysomy (≥3 copies of chromosomes in >40% of cells), high polysomy (≥4 copies of chromosomes in ≥40% of cells), and gene amplification (presence of gene clusters with a ratio of gene-to-chromosome of ≥2 per cell in ≥40% of cells or presence of small or non-enumerable clusters of the gene signal). On this basis patients were classified into two groups: FISH-negative (disomy and gains) and FISH-positive (high polysomy and/or gene amplification).

### PCR, RT-PCR and Mutation Analysis

Total RNA and genomic DNA were prepared as described [43], [44]. Q-RT-PCR and Q-PCR were performed using the Power SYBR Green PCR Master Mix in an ABI Prism 7300 thermocycler (Applied Biosystems, Foster City, CA, USA). cDNAs were synthesized from 1 µg of total RNA using QuantiTect Reverse Trascription (Qiagen, The Netherlands, Venlo). Normalization was performed to GAPDH mRNA content. The relative amounts of mRNA or DNA were calculated by the comparative cycle threshold (CT) method by Livak and Schmittgen [45]. Mutation analysis for PIK3CA using LightCycler was performed with DNA Master/Hybridization probes kit (Roche Molecular Biochemicals, Mannheim, Germany). Direct sequencing was performed using the BigDye v3.03 cycle sequencing kit (Applied Biosystems) in a capillary automatic sequencer (ABI PRISM 3100 Genetic Analyzer; Applied Biosystems). Protocols and primers for Q-PCR, Q-RT-PCR and sequencing KRAS (exons 2 and 3) and PIK3CA (exons 9 and 20) are reported in Supporting Information.

### Statistics

The association between the phosphorylation status of Akt and the expressed proteins or the clinicopathologic variables was evaluated by using the χ^2^ test. Q-PCR and Q-RT-PCR results were analyzed by two tailed Student’s t test or one-way ANOVA (GraphPad Software, Inc., La Jolla, CA, USA). The statistical significance threshold was set at a *P* value of.05 or less.

## Results

### AKT Activation in OC

As a read-out of PI3K/AKT signalling in OC from Italian patients we determined the phosphorylation status of aminoacid S473 of AKT1 (pAKT). pAKT was evaluated on TMAs containing duplicated core biopsies of 98 OC. As controls 50 matched normal samples (of which 3 tubes) were used. Patients’ clinico-pathological characteristics are described in Materials and Methods and summarized in Table S1.

**Figure 1:**
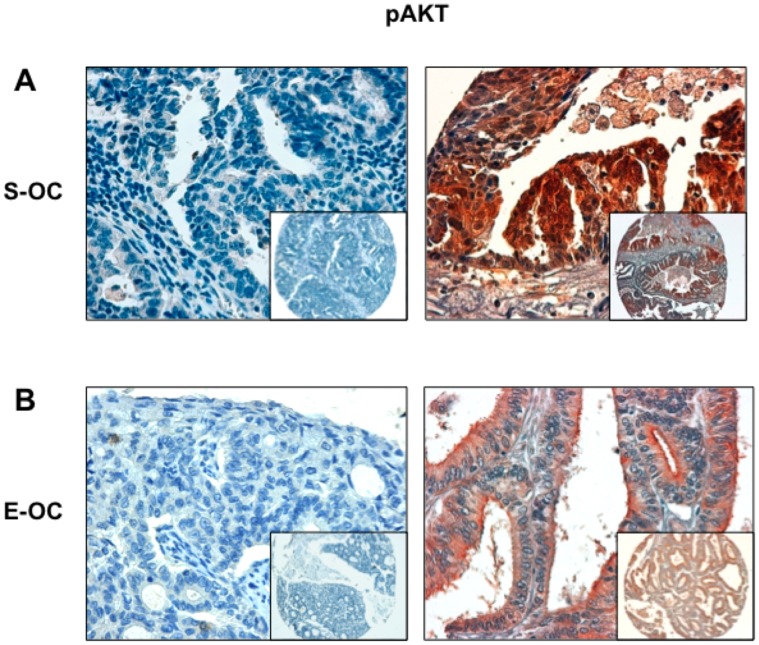
AKT pS473 immunostaining analysis in OC. A. Left: S-OC negative for pAKT phosphorylation; right: S-OC positive for pS473 phosphorylation. B. Left: E-OC negative for pAKT phosphorylation; right: E-OC positive for pS473 phosphorylation with apical enhancement. Magnification 40X. Magnification in the insets 10X.

Immunostaining analysis of TMAs failed to find AKT activation in 50 control tissues (Figure S1A). In contrast, AKT activation was observed in 73 out of 93 OC analysed and was significantly higher in cancer samples than in normal controls (Table 1; p<0.0001). pAKT staining was observed in 51/66 Serous Ovarian Carcinomas (S-OC) and in 14/16 Endometrioid Ovarian carcinomas (E-OC) (Table 1). See Figure 1 for representative stainings.

**Table 1:** AKT activation in OC.

	pAKT^a^			
	N°	Negative	Positive	*P value*
**Normal tissue**	50	50	0	<0.0001^b^
**Tumour tissue**	93	20	73	
**S-OC**	66	15	51	
**E-OC**	16	2	14	
**Mu-OC**	6	3	3	NS
**CC-OC**	3	0	3	
**M-OC**	2	0	2	

a Patients for which pAKT staining was available (N°).

b Normal vs Tumour Tissue.

**S-OC: S**erous **O**varian **C**arcinoma.

**E-OC: E**ndometrioid **O**varian **C**arcinoma.

**CC-OC: C**lear **C**ell **O**varian **C**arcinoma.

**Mu-OC: M**ucinous **O**varian **C**arcinoma.

**M-OC: M**ixed **O**varian **C**arcinoma.

**NS**: not significant.

We then correlated pAKT staining with the clinico-pathological parameters of patients (n = 98, median age 58 years old, range 21–86 years) and found a significant association between pAKT staining and the age of patients: Akt activation was more represented in patients ≥58 years old (p<0.05). On the contrary, we observed no significant association between pAKT staining and grade or FIGO stage of the disease (Table 2). See also Tables S2 and S3 for analysis of the correlation between pAKT and clinico-pathological parameters of patients with S-OC and E-OC, respectively, and Table S4 for a patient-by-patient list of pAKT status.

**Table 2:** Correlation between AKT activation (pAKT) and clinico-pathologic features of patients with OC.

	pAKT		
	Negative	Positive	*P value*
**Age** a			
<58y.o	14	32	0.038
≥58y.o	6	41	
**Tumour Grade** b			
G1	2	5	NS
G2	4	11	
G3	14	51	
**FIGO stage** c			
Stage I	4	16	NS
Stage II	2	7	
Stage III	13	47	
Stage IV	0	4	

a Patients for which pAKT staining was available (N = 93).

b Patients for which both Grade and pAKT staining were available (N = 87).

c Patients for which both Figo Stage and pAKT staining were available (N = 93).

**NS**: not significant.

### Mechanisms of AKT Activation in OC: AKT1 and AKT2

To investigate the molecular mechanisms leading to AKT activation in Italian patients affected by OC we performed a comprehensive analysis of the expression and/or the genetic status of AKT1 and AKT2 and their closest regulators (KRAS, PI3K and PTEN). Of the 98 cases on TMAs 257.1 and 257.2, 96 could be properly analysed for AKT1, 88 for AKT2, 89 for PTEN, 93 for p110α (PIK3CA) and 94 for p85α (PIK3R1) (Table S5). The evaluation criteria for the staining of each protein are reported in Materials and Methods. Accordingly, samples were classified as follows: negative (−), moderate (+) or high (++). In the case of PTEN staining samples were classified as positive (+), reduced (+/−) or negative (−).

**Figure 2:**
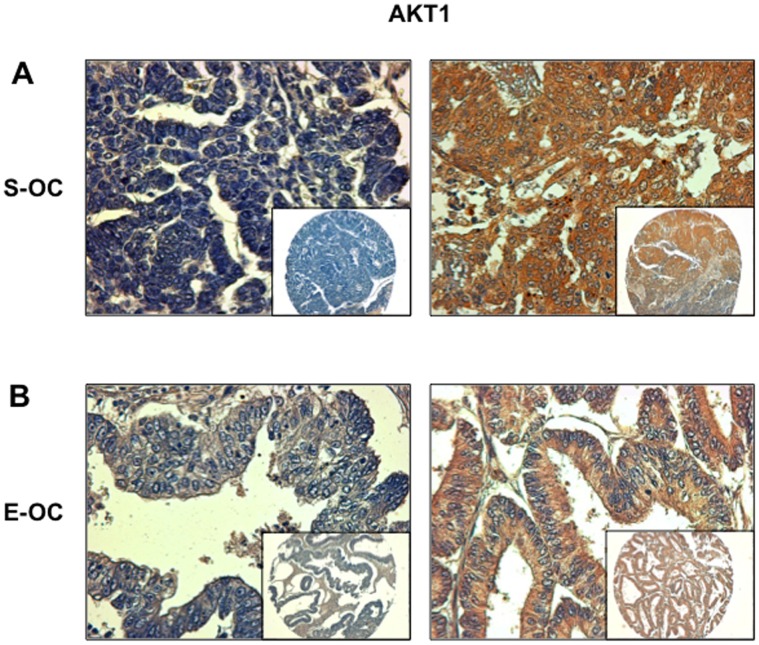
Immunostaining analysis of AKT1 in OC. A. Left: S-OC negative for AKT1 expression; right: S-OC positive for AKT1 expression. B. Left: E-OC negative for AKT1 expression; right: E-OC positive for AKT1 expression. Magnification 40X. Magnification of the insets 10X.

Analysis of TMAs 257.1 and 257.2 demonstrated that AKT1 presented moderate expression (+) in 32/96 cases and was frankly overexpressed (++) in 18/96 OC cases (∼19%) (Figure 2). Within AKT1 overexpressors 14 were S-OC and 3 were E-OC (Table S6). See also Figure S2A for representative staining of different levels of AKT1 expression in S-OC. Expectedly AKT1 overexpression – presented by the cases scoring (++) - was mirrored by AKT activation (17/18, 94%, Table 3).

**Table 3:** Correlation between AKT activation (pAKT) and expression of the different members of the PI3K pathway patients with OC.

		pAKT				
		Negative	Positive	N°		P value
**AKT1**	**Negative**	14	28	42		0.019
	**Moderate**	4	28	32		
	**High**	1	17	18		
**AKT2**	**Negative**	13	22	35		0.002
	**Moderate**	4	37	41		
	**High**	0	11	11		
**PIK3CA**	**Negative**	12	10	22		<0.001
	**Moderate**	0	7	7		
	**High**	8	55	63		
**PIK3R1**	**Negative**	6	6	12		0.001
	**Moderate**	6	7	13		
	**High**	8	59	67		
**PTEN**	**positive**	14	43	57		
	**reduced**	0	7	7		NS
	**negative**	2	22	24		

**NS**: not significant.

**Figure 3:**
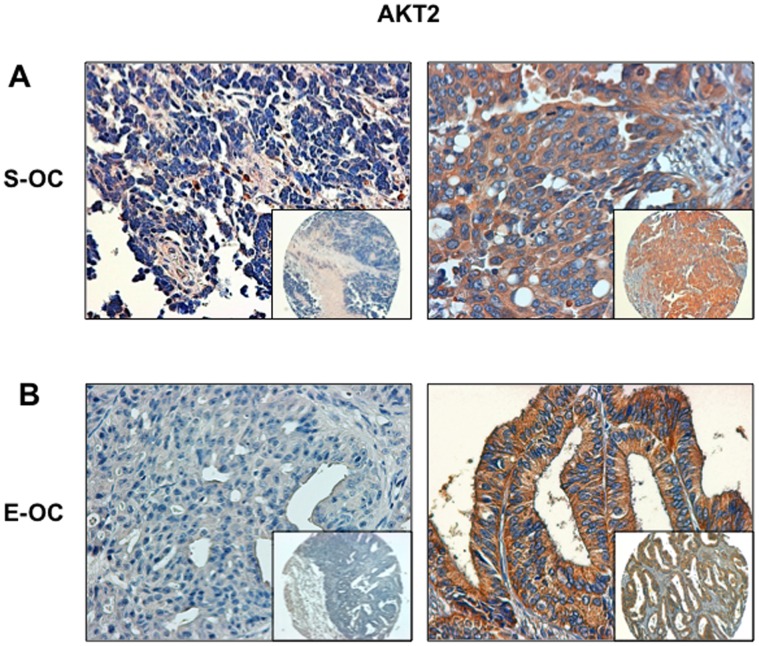
Immunostaining analysis of AKT2 in OC. A. Left: S-OC negative for AKT2 expression; right: S-OC positive for AKT2 expression. B. Left: E-OC negative for AKT2 expression; right: E-OC positive for AKT2 expression. Magnification 40X. Magnification of the insets 10X.

We then analysed expression of AKT2 in OC and found that it was moderately expressed in 41/88 specimens and overtly overexpressed in 11/88 specimens (12.5%) (Table S5 and Figure 3). See also Figure S2B for representative staining of AKT2 expression in S-OC. In the case of AKT2 all cases overexpressing AKT2 showed pAKT staining (Table 3). As with AKT1, AKT2 overexpression was similarly distributed among S-OC and E-OC [7 out of 61 S-OC (∼12%); 3 out of 16 E-OC (∼19%)] (Table S6).

As to the relationship between the expression of AKT1 and AKT2, we found that ∼14% OC showed an aberrant expression of only AKT1, ∼6% OC overexpressed AKT2 and ∼7% OC showed increased staining of both proteins (6/88 samples). On the other hand, 22 samples showed no sign of AKT1 or AKT2 overexpression. Notably, 32 out of 33 samples with aberrant staining for at least one of the proteins were positive for pAKT, while 12/22 (55%) negative specimens showed AKT activation.

### Mechanisms of AKT Activation in OC: PI3K

**Figure 4:**
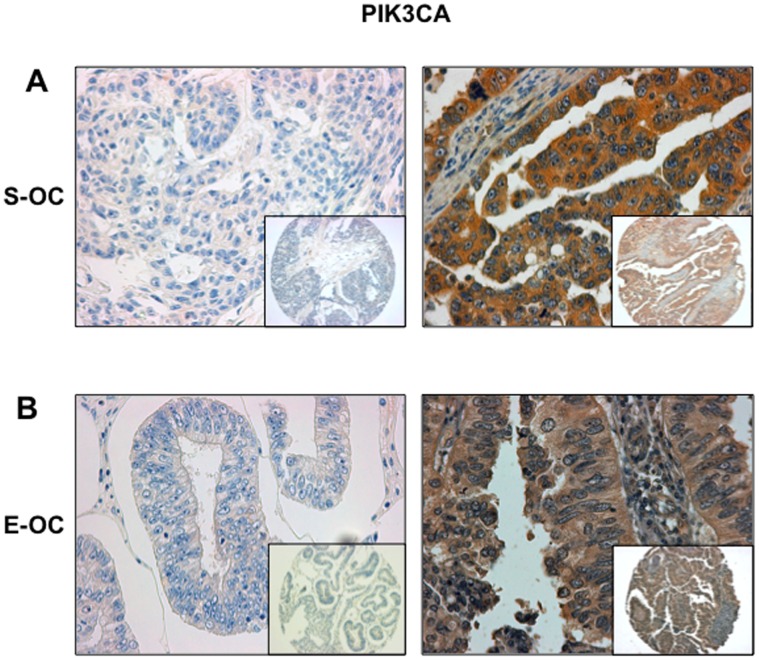
Immunostaining analysis of PIK3CA in OC. A. Left: S-OC negative for PIK3CA expression; right: S-OC positive for PIK3CA expression. B. Left: E-OC negative for PIK3CA expression; right: E-OC positive for PIK3CA expression. Magnification 40X. Magnification of the insets 10X.

Subsequently, we analysed the expression of both the catalytic and regulatory subunits of PI3K, p110α and p85α. Figure 4 and the Figures S2C and S3 show representative staining of PIK3CA e PIK3R1 in OC, respectively. We observed PIK3CA overexpression in 68% of OC (63/93) (Figure 4 and Table S5). Fifty out of 66 and 8/16 tumors overexpressing PIK3CA were S-OC (∼76%) and E-OC (50%), respectively (Table S6). As to the expression of p85α, we found that it was overexpressed in 69/94 patients (73%) (Figure S3 and Table S5). Fifty-one out of 66 samples (77%) overexpressing p85α were S-OC and 10 out of 16 (∼63%) were E-OC (Figure S3 and Table S6). Expectedly, OC overexpressing p110α (55/63; p<0.001) or p85α (59/67; p<0.001) presented significantly activated AKT (Table 3). Most samples simultaneously overexpress both the PI3K subunits (65/93, ∼70%; p = 0.001), suggesting that overexpression p110α is coordinated with that of p85α, leading to increased p110α-p85α heterodimer formation, and increased PI3K signalling [21].

### Mechanisms of AKT Activation in OC: FISH Analysis

**Figure 5:**
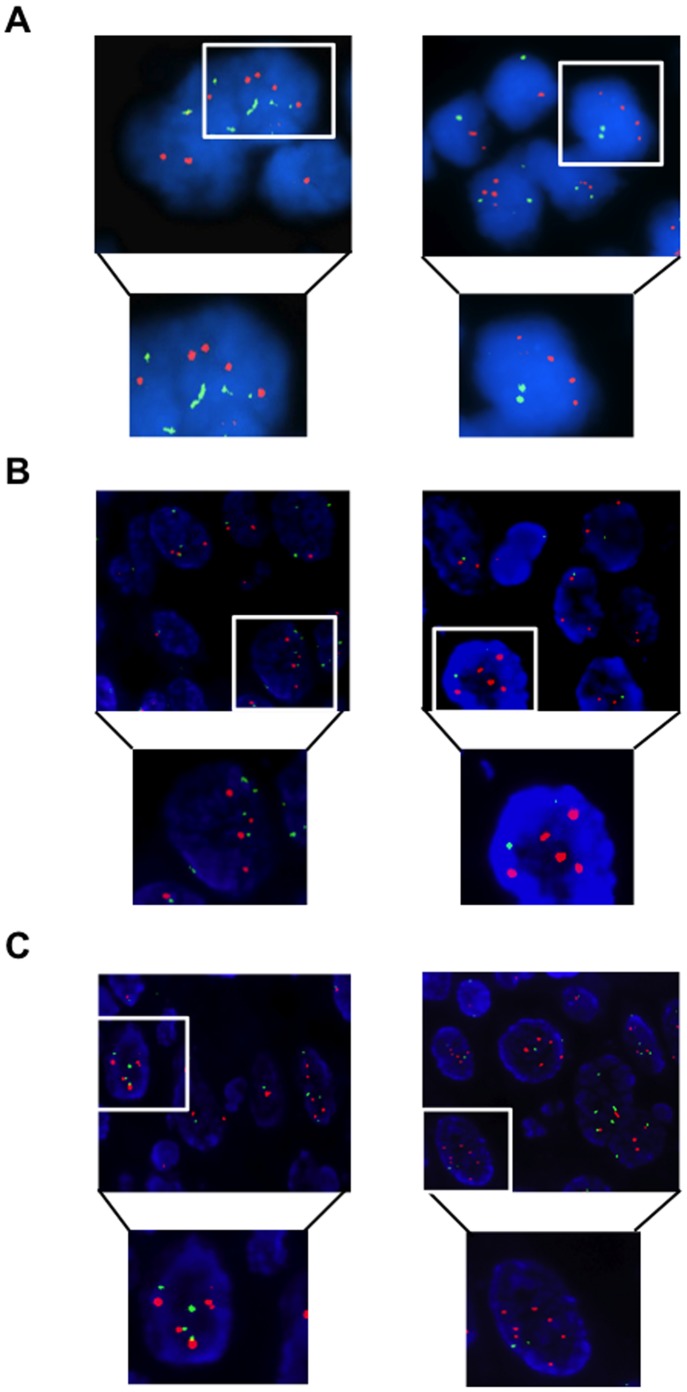
FISH analysis of AKT1, AKT2 and PIK3CA genes in OC. A. Dual-colour fluorescence in situ hybridization analysis of AKT1 gene copy number. AKT1 gene, red signals; chromosome 14 centromere, green signals. Left, OC with cells polyploidy for chromosome 14; right, OC with amplification of the AKT1 locus. Original magnification 100X. B. Dual-colour fluorescence in situ hybridization analysis of AKT2 gene copy number. AKT2 gene, red signals; chromosome region 19p13.1, green signals. Left, OC with cells polyploidy for chromosome 19; right, OC with amplification of the AKT2 locus. Original magnification 100X. C. Dual-colour fluorescence in situ hybridization analysis of PIK3CA gene copy number. PIK3CA gene, red signals; chromosome region 3p14.1, green signals. Left, OC with cells polyploidy for chromosome 3; right, OC with amplification of the PIK3CA locus. Original magnification 100X.

To determine the molecular mechanisms underling overexpression of AKT1, AKT2, PIK3CA and PIK3R1 we performed FISH and/or Q-PCR analysis in OC. See Materials and Methods for classification of tumours by FISH. We found that 16/55 OC (29%) presented copy number gain of the AKT1 gene at chromosome 14, of which 9 were high polysomy (>4 copies) and 7 focal amplification (Figure 5A). See Table S4 for a detailed list of genetic alterations detected in single OC patients. In the case of the gene encoding AKT2, we observed 10/36 OC (∼28%) with copy number gain at chromosome 19, of which 5 had high polysomy and 5 had focal amplification. See Figure 5B for a representative example. FISH analysis with chromosome 3q26.32 probes revealed the presence of an increase in the PIK3CA gene copy number in 25/84 cases (∼30%), all of which characterised by high polysomy, with 16 cases showing also focal amplification (Figure 5C). Of 25 cases showing copy number gains, 19 were S-OC, 3 was E-OC, 1 was CC-OC and 2 were M-OC.

On the other hand, the analysis of copy number variation indicated that one important mechanism that dysregulates AKT signalling in OC was the acquisition of an increased gene copy number – either amplification or high degree of polysomy. In fact, among the 63 tumors that overepressed PIK3CA, 11 presented amplification and 6 high polisomy (27%); among the 18 tumors that overepressed AKT1, 1 presented amplification and 4 high polisomy (∼28%) and among the 11 tumors that overepressed AKT2, 1 presented amplification and 1 high polisomy (18%).

For each gene under analysis most samples presenting high gene copy number showed high or moderate expression of the corresponding protein (8/16, 50%; 8/10, 80%; 20/25, 80%; for AKT1, AKT2 and PIK3CA, respectively). Moreover, most of FISH-positive OC resulted in the activation of AKT signalling. As summarized in Table 4, 20/24 cases that were FISH-positive for PIK3CA were positive for pAKT staining, 13/16 cases that were FISH-positive for AKT1 were positive for pAKT staining, 8/10 cases that were FISH-positive for AKT2 were positive for pAKT staining.

**Table 4:** Correlation between AKT activation and the presence of genetic alterations in PIK3CA, AKT1 and AKT2 in OC.

		pAKT		
		Negative	Positive	Total
**AKT1**	Negative^a^	11	27	38
	High copy^b^	3	13	16
**AKT2**	Negative^a^	5	21	26
	High copy^b^	2	8	10
**PIK3CA**	Negative^a^	11	46	57
	High copy^b^	4	20	24

a Low polisomy and negative samples for which pAKT staining was available.

b High polisomy and amplified samples for which pAKT staining was available.

**Figure 6:**
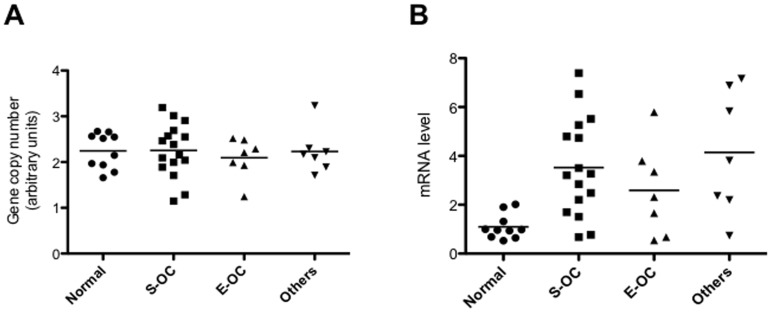
Analysis of the expression and of the gene copy number of PIK3R1. A. Q-PCR analysis of copy number of the PIK3R1 gene in normal ovarian tissue and OC. DNA from peripheral blood leukocytes (PBL) was used as control. PIK3R1 copy number in PBL was arbitrarly set as 2 (diploid value). B. PIK3R1 mRNA levels in normal ovarian tissue and OC. p = 0.006 (One-way Anova).

Given the high frequency of PIK3R1 overexpression in OC, we investigated the mechanisms underlying the observed overexpression of PIK3R1 protein. We used Q-PCR for analysis of PIK3R1 gene copy number and Q-RT-PCR for analysis of PIK3R1 mRNA in 30 representative primary OC (16 S-OC, 7 E-OC, and 7 others), that have been previously analyzed by immunostaining. The analysis of the genetic status of PIK3R1 by Q-PCR (Figure 6A) failed to reveal an increase in the copy number of the PIK3R1 gene in the 30 tumor analysed. Conversely, when analysed by Q-RT-PCR we found that 24 out of 30 OC (∼80%) showed increased mRNA levels compared with normal tissues (n = 10). Thirteen out of 16 were S-OC (81%), while five out of 7 were E-OC (71.4%). See Figure 6B. These results indicated that the increase in the levels of p85α observed in OC may be ascribed to increased transcription rate from the PIK3R1 gene promoter or to stabilization of the p85α protein by p110α but not to gene amplification.

### Mechanisms of AKT Activation in OC: Mutation Analysis

**Figure 7:**
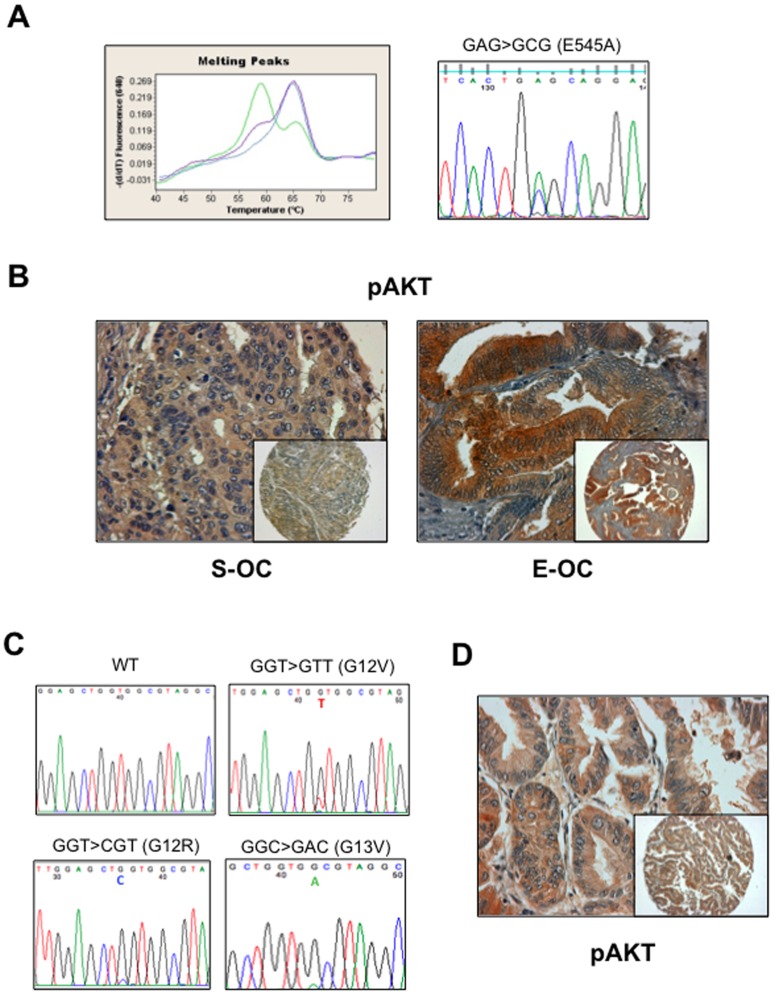
Mutation analysis of PIK3CA and KRAS genes in OC. A. Detection of mutations in PIK3CA by LightCycler (left) and direct sequencing (right). On the left, the negative derivative of the fluorescence (−dF/dT) versus temperature graph shows peaks with different Tm. The wild type sample showed a single Tm at 66°C. The heterozygous mutant sample showed an additional peak at 57°C. On the right, GAG→GCG transition in codon 545 of exon 9 inducing the substitution of a glutammic acid with an alanine (E545A). B. pAKT staining of a mutated S-OC (left) and E-OC (right). C. Point mutations in exon 2 of KRAS gene: GGT→GTT (G12V), GGT→CGT (G12R), GGT→ GAC (G13V). D. pAKT staining of a KRAS mutated sample (MU-6).

We also investigated the occurrence of mutations in the PI3KCA or KRAS genes in OC. Because the vast majority of PIK3CA gene mutations in cancer were reported in exons 9 and 20, we focused our mutation analysis on these exons [46]. Mutation detection was performed on the LightCycler (Roche) and confirmed by direct DNA sequencing (see Figure 7A). We observed the missense mutation (GAG1633->ACG that lead to the amino acid change E545A) in the PIK3CA gene in 5 cases out of 33 analysed (12%) of which 3 S-OC and 2 were E-OC. In agreement with previous findings PI3KCA mutations were observed in E-OC [23]. In the case of PIK3CA, mutations and gene amplification were apparently mutually exclusive, though, due to the limitedness of the sample under analysis, this correlation did not reach statistical significance (p = 0.05). Interestingly, 4 tumors with mutations in PIK3CA were positive for pAKT, except for one S-OC specimen. See Figure 7B.

Moreover, a total of 31 tumours were successfully analyzed for the presence of mutations in the gene encoding KRAS. Overall, mutations were found in 3 samples (∼10%). Three different mutations were detected in the OC analysed: G12V (GGT->GTT); G12R (GGT->CGT); G13D (GGC->GAC). The G12V mutation was found in one S-OC patient, while the G12R and G13V mutations were detected in Mu-OC. The specific nucleotide changes and the corresponding amino acid substitutions are shown in Figure 7C. The overall KRAS mutation frequency in this cohort of OC (∼10%) was in agreement with that described in previous works (10–15%) [47], [48]. KRAS mutations were observed prevalently in the Mu-OC subgroup [49], [50] and in a low grade S-OC (S53) [51]. KRAS mutation was reflected into AKT activation in 2 tumors out of three (Figure 7D). See Table S4 for details.

### Mechanisms of AKT Activation in OC: PTEN

**Figure 8:**
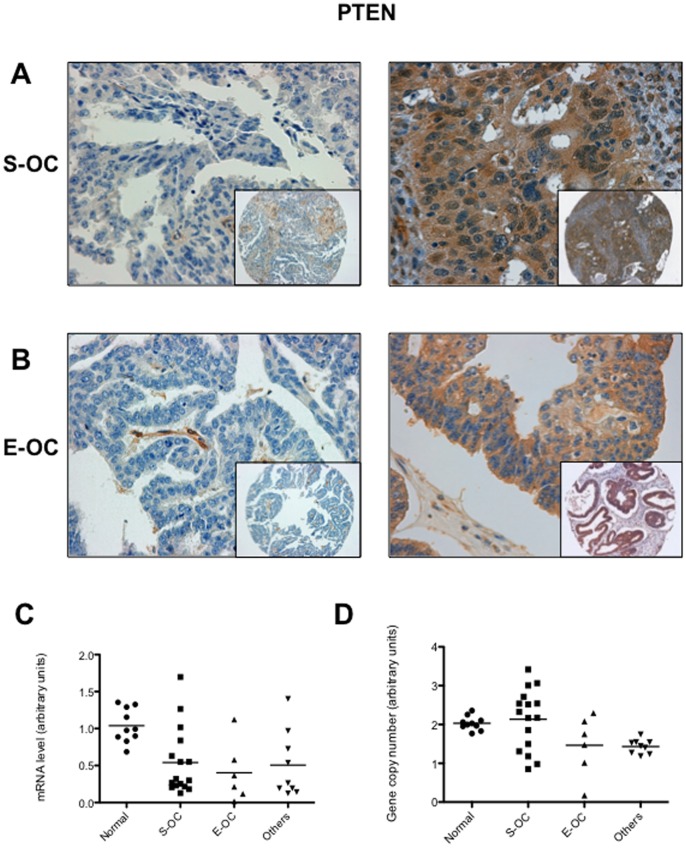
Immunostaining and gene copy number analysis of PTEN in OC. A. Left: S-OC negative for PTEN expression; right: S-OC positive for PTEN expression. B. Left: E-OC negative for PTEN expression; right: E-OC positive for PTEN expression. Magnification 40X. Magnification of the insets 10X. C. Q-RT PCR of PTEN mRNA expression in normal ovarian tissues and OC. D. Q-PCR analysis of PTEN gene copy number in normal ovarian tissues and OC. DNA from peripheral blood leukocytes (PBL) was used as reference. PTEN copy number in PBL was set arbitrarily as 2.

Patients accrued for this study were also characterised for PTEN expression. Complete loss of PTEN protein was observed in 24 of 89 (∼27%) OC; reduction of PTEN expression was observed in 7 additional cases (Figure 8A and 8B; Table S5). In agreement with previous studies, PTEN loss was less frequent in S-OC (15/63, ∼24%) than in others histotypes (9/26, ∼35%): 4/14 E-OC, 2/6 Mu-OC, 2/4 CC-OC and 1/2 M-OC (See Tables S4 and S6) [52]. However, AKT activation was significantly associated with the loss and/or reduction of PTEN expression only in S-OC (n = 63; p = 0.019) (Table S7).

We used quantitative RT-PCR analysis of normal ovarian tissue samples (*n = *10) and 31 representative primary OC (16 S-OC, 6 E-OC, and 9 others), that have been analyzed by immunostaining, to investigate whether the loss of PTEN protein observed in the TMA studies occurred through mechanisms that dysregulate its mRNA transcription (Figure 8C). By matching the quantitative RT-PCR analysis of PTEN mRNA with the protein analysis performed on TMAs, we observed poor correspondence between loss of PTEN mRNA and loss of the corresponding protein. Using a cut-off of 0.7 (arbitrary unit) as lower limit for normal PTEN mRNA level, we found that 23 OC showed reduced mRNA levels; however, only 7 out of them also showed consistently loss of PTEN protein. On the other hand, 2 out of 8 samples, that showed mRNA expression comparable with normal tissue, had lost protein expression suggesting also the existence of a post-translational mechanism.

We also investigated the genetic status of PTEN by Q-PCR on the same group of samples analysed by Q-RT-PCR (Figure 8D). The average value of PTEN gene in normal tissues was similar to the PBL’s value that had been set arbitrarily as 2. We used a cut-off of 1.3 (arbitrary unit) as lower limit for PTEN biallelic status and 0.3 as lower limit for monoallelic status. We found that 3 S-OC, 2 Mu-OC and one E-OC and M-OC showed monoalellic loss of PTEN gene whereas one E-OC showed biallelic loss of PTEN gene, for a total of 8 samples out of 31 analysed (26%). Importantly, most of the samples that showed monoallelic or biallelic deletion of PTEN gene (6/8) presented drastically reduced or absent mRNA and PTEN protein. Moreover 7/8 samples with monoallelic or biallelic deletion of PTEN gene showed AKT activation. Altogether these results indicate that multiple mechanisms are responsible for PTEN loss during OC carcinogenesis.

It is of note that from the analysis of TMAs we found that aberrant expression of more than a single gene within the PI3K pathway (PTEN loss, overexpression of AKT1, AKT2 or p110α, respectively) was observed only in tumours showing AKT activation. In particular, PTEN loss was mutually exclusive with increased AKT2 expression but not with increased expression of AKT1 or PIK3CA and that >50% of tumors overexpressing PIK3CA presented a second molecular alteration (see Table 5 and Tables S8–S10).

**Table 5:** Altered expression of genes within PI3K pathway (AKT2, AKT2, PTEN, PIK3CA) and AKT activation (pAKT) in OC.

Alteration	pAKT negative(N = 20)	pAKT positive(N = 73)
AKT1^a^	1	1
AKT2^b^	0	0
PIK3CA^c^	8	23
PTEN^d^	0	4
AKT1, PTEN	0	1
AKT2, PTEN	0	0
PIK3CA, PTEN	0	14
AKT1, AKT2	0	2
PIK3CA, AKT1	0	6
PIK3CA, AKT2	0	5
AKT1, AKT2, PTEN	0	0
AKT1, PIK3CA, PTEN	0	3
AKT2, PIK3CA, PTEN	0	0
AKT1, AKT2, PIK3CA	0	4
AKT1, AKT2, PIK3CA, PTEN	0	0

a High AKT1 expression as defined in the manuscript.

b High AKT2 expression as defined in the manuscript.

c High PIK3CA expression as defined in the manuscript.

d PTEN loss as defined in the manuscript.

### Analysis of Pathways Activated Downstream PI3K in OC: mTOR and SGK3

AKT is the most important effector of PI3K signalling, and is a key regulator of a variety of proteins involved in cell proliferation, metabolism, invasion, migration, and apoptosis that include mTOR (mammalian target of rapamycin), GSK3, and forkhead transcription factors [53]. In particular, mTOR is a critical component of the PI3K/AKT pathway that activates protein synthesis and cell proliferation [54].

Therefore we investigated whether the activation of the PI3K/AKT pathway observed in OC in this study was associated with activation of mTOR or of two well characterized downstream targets such 4EBP1 and p70 S6 kinase (S6K1). To this aim, we studied the activation status of mTOR, 4EBP1, S6K1 and its substrate S6 in immunostaining on TMAs, by use of phospho-specific antibodies (anti-phospho-mTOR, Ser2448; anti-phospho-S6K1, Thr389; anti-phospho-4EBP1, Thr37/46; anti-phospho-S6, Ser235/236), and correlated them with AKT activation and/or PIK3CA overexpression.

We found that the protein encoding mTOR was expressed in all normal and cancer-derived samples (data not shown) whereas mTOR phosphorylation was detected at high level in 46/92 OC (50%), of which 33 were S-OC e 9 were E-OC. Similarly, S6K1 protein was expressed in all normal and cancer-derived samples (data not shown) whereas S6K1 phosphorylation was detected at high level in 65/91 OC (71.5%), of which 47 were S-OC e 11 were E-OC.

Finally, 4EBP1 was phosphorylated in 52 out of 92 OC (56%) whereas S6 was significantly phosphorylated in 36 out of 91 OC (40%). As with mTOR and S6K1, both S6 and 4EBP1 proteins were constitutively expressed in tumors (data not shown).

**Figure 9:**
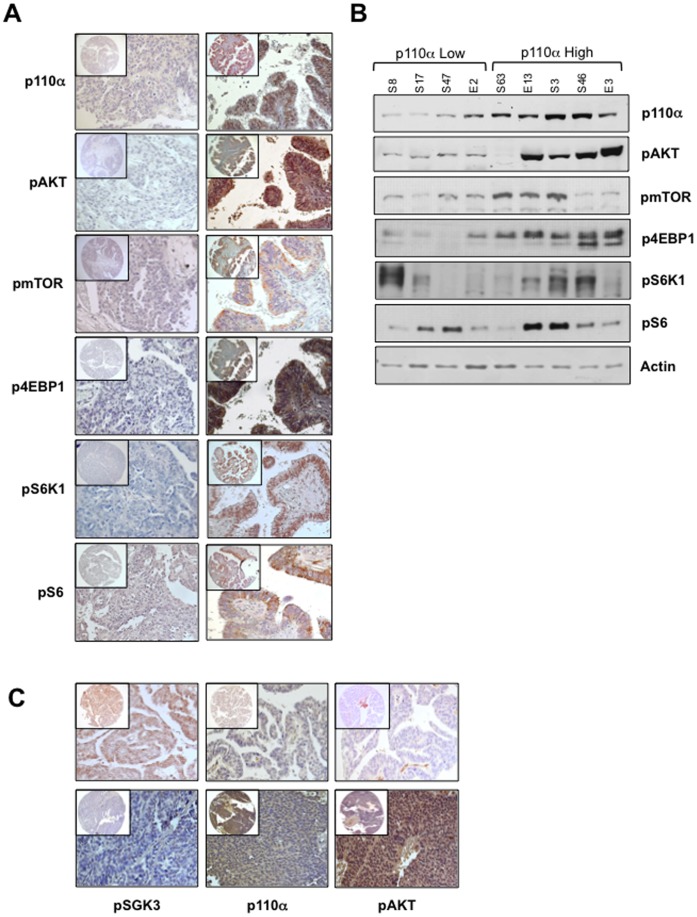
Analysis of pathways activated downstream PI3K in OC: mTOR and SGK3. A. Immunostaining analysis of phosphorylated AKT, mTOR, S6K1, S6, and 4EBP1 in two representative formalin-fixed samples: left, p110α low (S17) and right, p110α high (S46). B. Protein from fresh-frozen samples were assayed by Western blot with the indicated antibodies. Samples S8, S17, S47, E2: low p110α expression; samples S63, E13: PIK3CA mutation; samples S63, S46, E3: p110α over-expression. Actin expression was used as a control for protein quality and loading. C. Representative pSGK3 immunostaining of pSGK3-positive (upper left panel) and pSGK3-negative (lower left panel) samples. Middle panels, immunostaining analysis of pAKT; right panels, immunostaining analysis of p110α of the same samples. Magnification 40X. Magnification of the insets 10X.

To determine whether the mTOR/S6K1/4EBP1 pathway contributed to aberrant PI3K signalling observed in OC we correlated the phosphorylation of mTOR, S6K1, 4EBP1, and S6 with PIK3CA overexpression and/or AKT activation. We found that several OC that were positive for pmTOR or pS6K1 were also positive for either pAKT (34/44 and 48/62, respectively) or p110α (33/44 and 44/62, respectively) staining, though no significant correlation was observed among these proteins (Table 6). Conversely, phosphorylation of 4EBP1 was correlated with p110α overexpression, AKT activation (pAKT) and mTOR activation (pmTOR) (Table 6) and, similarly, phosphorylation of S6 presented a significant correlation with AKT activation (pAKT) and mTOR activation (pmTOR) (Table 6). Immunostaining data were confirmed by Western blot analysis of a representative group of freshly-frozen OC (Figure 9) and on a selected subset of OC cell lines (Figure S4).

**Table 6:** Correlation between the different members of the mTOR pathway with overexpression/mutation of PIK3CA and activation of AKT (pAKT) or mTOR (pmTOR) in patients with OC.

		PIK3CA			pAKT			pmTOR		
		Neg	High	*P*	Neg	Pos	*P*	Neg	Pos	*P*
**pmTOR**	**Neg**	10	36	NS	9	37	NS	/	/	/
	**Pos**	11	33		10	34		/	/	
**p4EBP1**	**Neg**	13	26	0.05	12	26	0.04	25	13	0.01
	**Pos**	8	43		7	45		20	32	
**pS6K1**	**Neg**	4	20	NS	3	21	NS	17	7	0.01
	**Pos**	18	44		14	48		25	36	
**pS6**	**Neg**	13	40	NS	16	37	0.006	24	30	NS
	**Pos**	8	26		2	32		17	15	
**pSGK3**	**Neg**	16	41	NS	12	44	NS	30	25	NS
	**Pos**	7	24		7	25		13	19	

**NS**: not significant.

These results suggest that the mTOR/S6K1/4EBP1 pathway contribute to aberrant PI3K signalling and AKT activation in OC.

On the other hand, recent studies uncovered an AKT-independent signalling pathway in PIK3CA mutant cancer cell with low AKT activation that involved PDK1-dependent activation of SGK3 [55]. Therefore, to understand whether SKG3 could mediate PI3K signalling in tumors that presented low AKT activation in the setting of high PIK3CA expression, we analyzed SGK3 activation using phosphorylation specific antibody (Thr320). We found that that 33/92 OC (36%) were positive for phospho-SGK3 staining though phosphorylation of SGK3 did not correlate with p110α overexpression or AKT activation (see Table 6 and Figure 9C). On the other hand, activated pSGK3 was detected only 3 out of 9 OC that were positive for p110α and negative for pAKT, showing a frequency of SGK3 activation similar to OC positive for p110α and positive for AKT. Altogether, these results suggest that, at least in the OC analysed here, aberrant PI3K activity is mediated by the canonical AKT-dependent mTOR/S6K1/4EBP1 pathway but not by the novel AKT-independent activation of SGK3.

### Analysis of Pathways Activated Downstream PI3K in OC: Transcription Factors

**Figure 10:**
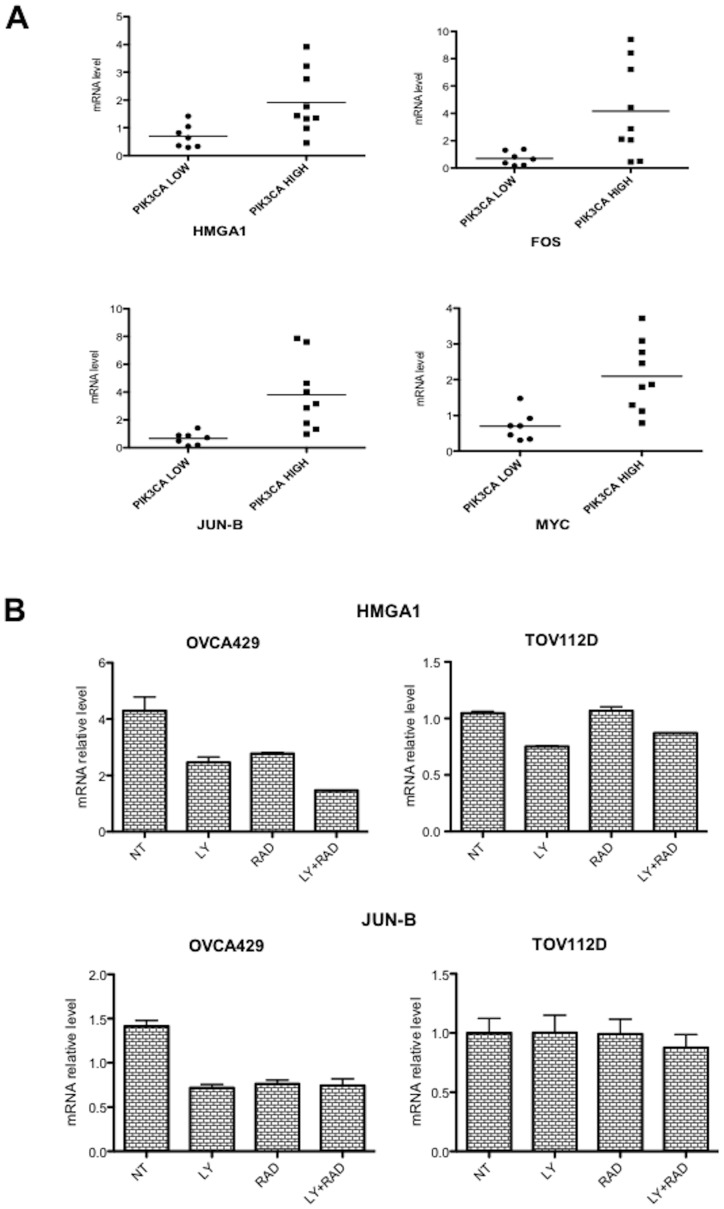
Analysis of pathways activated downstream PI3K in OC: HMGA1, JUN-B, FOS, MYC. A. Q-RT-PCR analysis of OCs with low or high expression of PIK3CA. B. Q-RT-PCR analysis of HMGA1 (top) and JUN-B (bottom) in OVCA429 (left) and TOV112D (right) treated with LY294002 (LY, 20 µM), RAD001 (RAD, 20nM) or a combination thereof for 24h. Values of mRNA are expressed as relative values using as standard the value of normal ovarian epithelial cells (IOSE 398).

We have recently demonstrated that aberrant PI3K signalling in Non-Small Cell Lung Cancer cells induces the mRNA expression of oncogenic transcription factors (HMGA1, JUN-B, FOS and MYC). Thus, to further extend the analysis of the pathways acting downstream PI3K and/or AKT, we investigated whether overexpression of PIK3CA affected the expression of these transcription factors also in OC. To this aim, we performed Q-RT-PCR analysis of HMGA1, JUN-B, FOS and MYC in a representative number of samples that were classified into two groups: low expressors/non-mutated p110α (n = 7) or high expressors/mutated p110α (n = 9). As shown in Figure 10A, we found that the group of PIK3CA overexpressors showed an increased average expression of the mRNAs encoding HMGA1 (p = 0.02), JUN-B (p = 0.006), FOS (p = 0.02), and MYC (p = 0.003), compared with the group containing low expressors samples. The average values for PIK3CA low-expressors were 0.699±0.16 for HMGA1, 0.669±0.17 for JUN-B, 0.696±0.18 for FOS, 0.702±0.154 for MYC; conversely, the average values for PIK3CA overexpressors were 1.912±0.379 for HMGA1, 3.802±0.843 for JUN-B, 4.2±1.135 for FOS, 2.1±0.326 for MYC. We further confirmed this observation by RT-PCR analysis of selected OC cell lines (OVCA429, TOV112D) that had been treated with PI3K or mTOR inhibitors (LY294002 and RAD001, respectively). Of note that both inhibitors reduced the levels of all 4 transcription factors in OVCA429 but not in TOV112D (Figure 10B), suggesting that mTOR was implicated in the PI3K-dependent control of the expression of transcription factors.

## Discussion

We report a comprehensive analysis of the contribution of the different members of PI3K/AKT pathway to AKT deregulation and to the development of ovarian cancer in a cohort of Italian OC patients. We report that in the cohort of OC patients studied here, the PI3K/AKT pathway is activated in ∼79% of the cases (73/93) and was detected more frequently in the elderly (>58 years old; n = 93, p<0.05). This value is in agreement with the average degree of PI3K/AKT activation as reviewed in Bast [12] but apparently differs from data reported in previous studies on cohorts of Norwegian (51.6%) [56] or Middle Eastern OC patients (52.1%) [42], thus highlighting possible ethnical and/or geographical differences.

Immunoistochemical studies have suggested that AKT activation is common in high-grade, late stage S-OC [20], [18] and is associated with resistance to cytotoxic therapies [17]. However, at difference with some of these previous studies, in Italian OC patient AKT S473 phosphorylation was not associated to advanced disease or higher grade. The difference in AKT activation between FIGO stages I-II and FIGO stages III-IV was not statistically significant, possibly due to the relative paucity of FIGO stages I-II in our analysis. Moreover, no gross difference in AKT activation was observed with regard to histological subtypes (77% in S-OC; 87% in E-OC) and grade (71% in low-grade; ∼78% in high-grade). Altogether these results suggest that deregulation of the PI3K/AKT signalling occurs early during the tumorigenic process in the ovary and represents a common event for the different molecular routes through which OCs develop.

A second finding of this study was that several OCs simultaneously over-express the catalytic (PIK3CA) and the regulatory (PIK3R1) subunits of PI3K (∼57%) or present activating mutations in the gene encoding PIK3CA (∼12%), implying that the deregulated expression and/or activity of this enzyme represents a crucial oncogenic event during cancer development in the ovary. Accordingly, aberrant activation of p110α in OCs – either due to overexpression or mutations - was reflected into AKT activation and, as a whole, represented the major determinant of AKT activation in this cohort of patients. These results extend to the Italian cohort of patients the observation that PI3K is an important oncogene for the development and progression of OC [21], [42], [57]. Less frequent alterations comprised PTEN loss (∼27%), overexpression of AKT1 (∼19%) or of AKT2 (∼14%) and mutations in the KRAS genes (10%). Conversely, OCs analysed in this study were negative for mutations in AKT1 or AKT2, confirming previous reports [58]. As to PTEN inactivation, somatic inactivating mutations of PTEN are rather rare in OCs, having been described preferentially in CC-OCs and in low-grade E-OCs [59], [60]. Accordingly, we did not detect PTEN mutations, possibly because of the low number of CC-OCs analysed (data not shown). Conversely, we observed a large reduction in PTEN protein expression in approximately 27% of cases including high-grade S-OCs, E-OCs and Mu-OCs. A third issue to discuss of our results is that the type and/or the position of the alterations identified within the PI3K/AKT pathway apparently have different strength and effects [61]–[63]. In fact, whereas mutations of PIK3CA are mutually exclusive with other genetic alterations, the majority of OCs under analysis here (n = 35) presented alterations in more than one gene, suggesting that the different genetic alterations of the PI3K/AKT pathway in OCs are not functionally redundant. In fact, activating mutations in PIK3CA are mutually exclusive with amplification or other forms of copy number gains (except for patient S56), suggesting that they are sufficient not only to activate AKT but also to drive ovarian tumorigenesis independently, as demonstrated in other cohorts [42]. On the other hand, more than half OC that overexpressed PIK3CA presented two or more alterations (14 OCs simultaneously overexpressed PIK3CA and had lost PTEN, 6 OCs overexpressed both PIK3CA and AKT1, and 5 OCs overexpressed both PIK3CA and AKT2). Also KRAS mutations were detected in a contest of PTEN loss (Mu6, Mu7). At difference with the OCs that presented a single alteration, all OCs with at least 2 alterations showed strong AKT activation. These findings are reminiscent of breast or endometrial cancer, in which PIK3CA mutations are frequently detected in the setting of low PTEN expression or mutations [64], [65], and suggest that p110α over-expression alone may not be sufficient to activate AKT signalling and hence requires other alterations to be fully oncogenic in OC. The functional effects of mutant or amplified PIK3CA in OC cells have defined through adoptive expression of mutant p110α or by silencing endogenous activated PIK3CA alleles [66]–[68]. Collectively, the data available indicate that PI3K signalling is required both *in vitro* and in vivo. However, our results in the human indicate that both PI3K and PTEN act in concert with other oncogenic hits to promote malignant transformation of ovarian epithelial cells and recent data in the mouse have demonstrated that the overexpression of activated PIK3CA or the loss of PTEN in the mouse ovarian surface epithelium do not lead to tumor formation [69].

As to the mechanisms whereby expression and/or activity of the genes under analysis are dysregulated in OC, we found that, in many OC, the abnormal expression/activity of the genes within the PI3K/AKT pathway could be ascribed to underlying genetic alterations. PIK3CA presents focal amplification at 3q26.3 or high polysomy of the chromosome 3 in ∼30% of primary OCs while AKT1 and AKT2 present increased copy number in ∼30 and ∼28% of OCs. Expectedly, most samples presenting high gene copy number of PIK3CA, AKT1 and AKT2 showed high or moderate expression of the corresponding proteins (80%; 50%; 80% for PIK3CA, AKT1 and AKT2, respectively), with most FISH-positive OC resulting in the activation of AKT signalling.

As to PTEN, the majority of tumors showed loss of PTEN protein and consistently reduced mRNA levels, suggesting that the major mechanism of PTEN inactivation occurs at the transcriptional level that in some cases (about 30%) may be ascribed to monoalellic or biallelic loss at the PTEN locus on chromosome 10. However, the finding of tumors with high mRNA and low protein suggests the existence of a post-translational mechanism of PTEN inactivation in OCs as demonstrated in lung cancer. At least two potential mechanisms can account for the observed discrepancy between PTEN mRNA and protein levels. The first is an increased turnover of the PTEN protein due to overxpression of the ubiquitin ligase NEDD4-1 as recently demonstrated to occur in NSCLC [70]. A second mechanism that may account for reduced levels of PTEN protein in the presence of normal mRNA level is the potential effects of oncogenic miRNAs. Accordingly, Yang and coworkers have reported that miR-214 blocks PTEN translation leading to AKT activation drug resistance [71].

A final observation deriving from our results is that the aberrant PI3K activity observed in OC, is apparently mediated by activation of the downstream AKT-dependent mTOR/S6K1/4EBP1 canonical pathway and by regulation of expression of oncogenic transcription factors that include HMGA1, JUN-B, FOS and MYC but not by AKT-independent activation of SGK3.

In conclusion, the results reported in this manuscript indicate that the different genetic alterations of the PI3K/AKT pathway in OC are not functionally redundant and that the type or the position of the alteration within the PI3K/AKT pathway may influence mechanisms and effects of pathway deregulation. In particular, PI3KCA over-expression occurs, through gene copy number gains, at a much higher frequency in ovarian cancer than do activating mutations, apparently representing the major determinant of AKT activation in OC. However, p110α over-expression alone is not apparently sufficient to activate AKT signalling in OCs and hence may require other alterations to be fully oncogenic.

## Supporting Information

Figure S1 **Expression** **of pAKT, AKT1, AKT2, PIK3CA, PIK3R1, and PTEN in tubal epithelium.** A. pAKT. B. AKT1. C. AKT2. D. PIK3CA. E. PIK3R1. F. PTEN. Magnification 40X. Magnification of the insets 10X.
(TIF)

Figure S2 **Immunostaining analysis of AKT1, AKT2, PIK3CA and PTEN in OC.** A. Different degree of AKT1 expression in OC. From left to right: negative (−), moderate (+) and high (++) expression. B. Different degree of AKT2 expression in OC. From left to right: negative (−), moderate (+) and high (++) expression. C. Different degree of PI3KCA expression in OC. From left to right: negative (−), moderate (+) and high (++) expression. D. Different degree of PTEN expression in OC. From left to right: negative (−), reduced (−/+) and positive (+).Magnification 40X. Magnification of the insets 10X.
(TIF)

Figure S3 **Immunostaining analysis of PIK3R1 in OC.** A. Different degree of PIK3R1 expression in S-OC. From left to right: negative (−), moderate (+) and high (++) expression. B. Different degree of PIK3R1 expression in E-OC. From left to right: negative (−), moderate (+) and high (++) expression. Magnification 40X. Magnification of the insets 10X.
(TIF)

Figure S4 **Analysis of pathways activated downstream PI3K in OC: mTOR and SGK3.** Western blot analysis of phosphorylated AKT, mTOR, S6K1, S6 and 4EBP1 in ovarian cancer cell lines with absent (lanes 1–5) or present (lanes 6–8) genetic alterations that activate the PI3K/AKT pathway.
(TIF)

Table S1 **Samples distribution among hystotypes and clinico-pathological subclasses.**
(DOC)

Table S2 **Correlation between AKT activation and clinico-pathologic features of S-OC patients.**
(DOC)

Table S3 **Correlation between AKT activation and clinico-pathologic features of E-OC patients.**
(DOC)

Table S4 **Patient-by-patient list of the genetic alterations observed in OC patients.**
(DOC)

Table S5 **Immunostaining evaluation of the members of the PI3K/AKT pathway.**
(DOC)

Table S6 **Immunostainng of the members of the PIK3/AKT pathway in different OC histotypes.**
(DOC)

Table S7 **Correlation between AKT activation (pAKT) and the expression of the different members of the PI3K/AKT pathway in S-OC patients.**
(DOC)

Table S8 **Correlation between AKT activation (pAKT) and the expression of the different members of the PI3K/AKT pathway in E-OC patients.**
(DOC)

Table S9 **Correlation between alterations in the expression of PTEN, PIK3CA, AKT1 and AKT2 and pAKT status in S-OC.**
(DOC)

Table S10 **Correlation between alterations in the expression of PTEN, PIK3CA, AKT1 and AKT2 and pAKT status in E-OC.**
(DOC)

Materials and Methods S1 **PIK3CA mutational analysis (Light cycler).** Cell lines and treatment. Primer sequences.
(DOC)
