# Supplementary figures and images for: Multiple Genetic Alterations within the PI3K Pathway Are Responsible for AKT Activation in Patients with Ovarian Carcinoma
Source: PLoS One. 2013 Feb 7;8(2):e55362. doi: 10.1371/journal.pone.0055362 (PMC3567053; doi:10.1371/journal.pone.0055362)

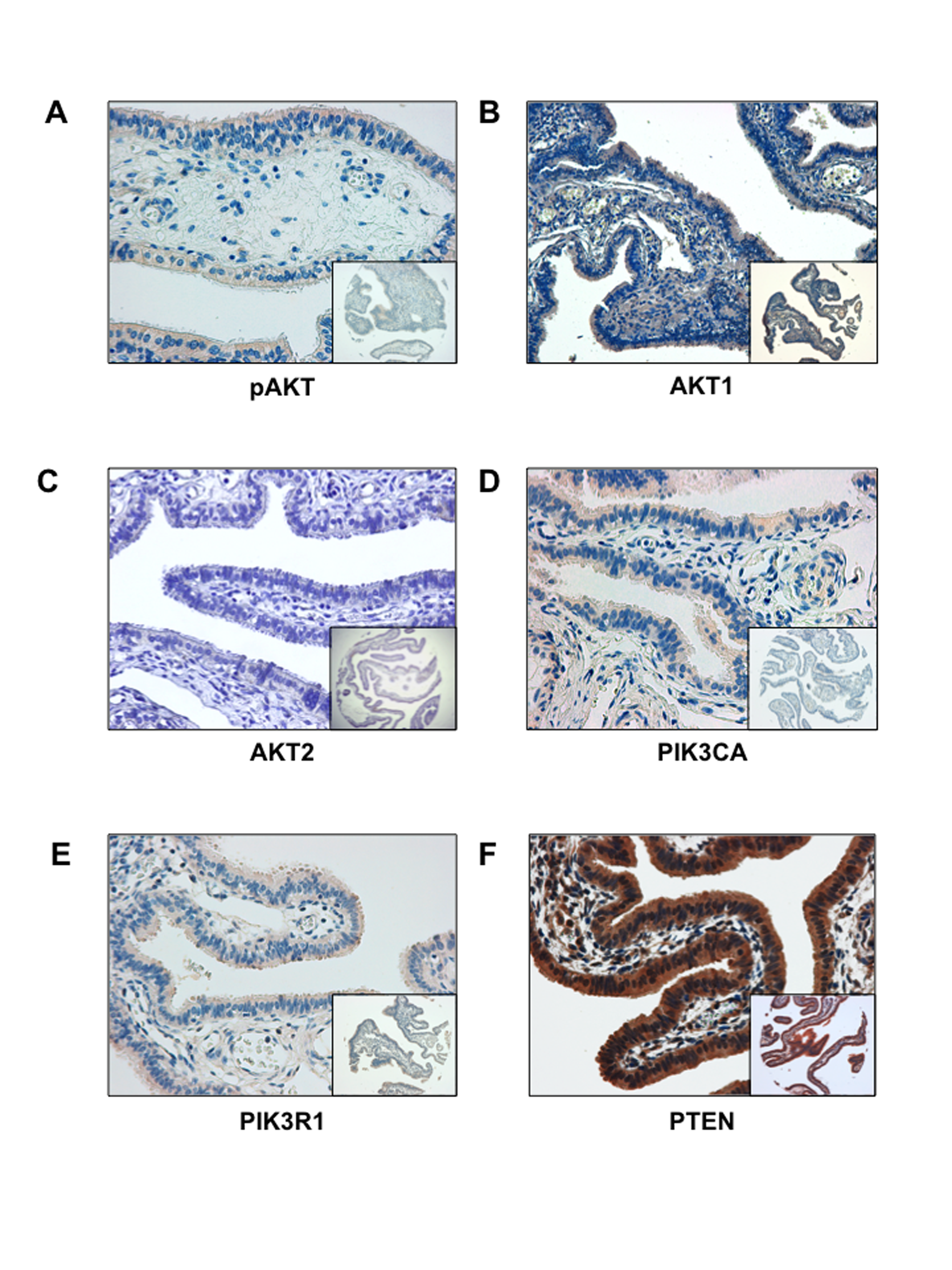

Supplement: Figure S1 — Expression of pAKT, AKT1, AKT2, PIK3CA, PIK3R1, and PTEN in tubal epithelium. A. pAKT. B. AKT1. C. AKT2. D. PIK3CA. E. PIK3R1. F. PTEN. Magnification 40X. Magnification of the insets 10X. (TIF) [file pone.0055362.s001.tif]

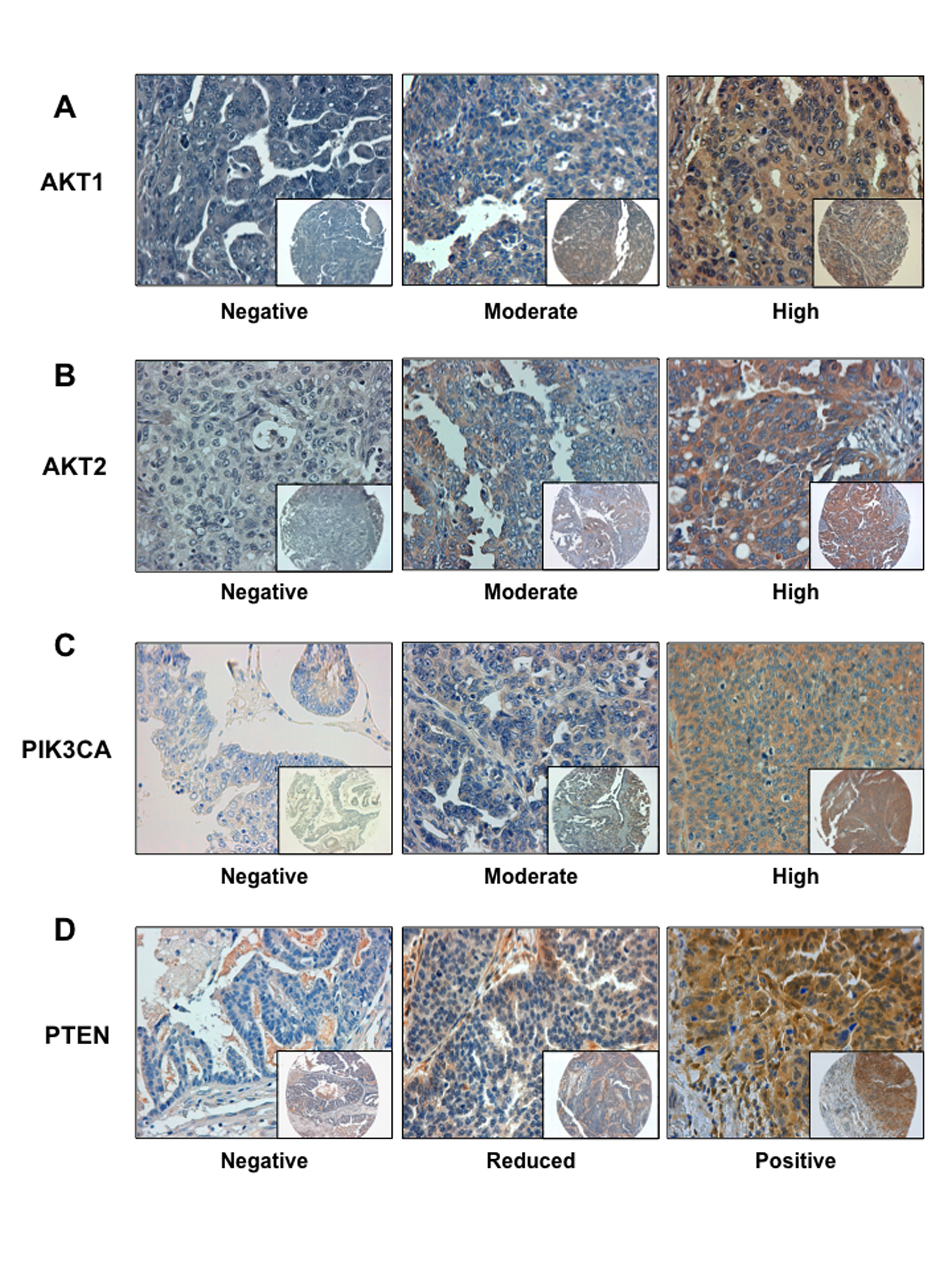

Supplement: Figure S2 — Immunostaining analysis of AKT1, AKT2, PIK3CA and PTEN in OC. A. Different degree of AKT1 expression in OC. From left to right: negative (−), moderate (+) and high (++) expression. B. Different degree of AKT2 expression in OC. From left to right: negative (−), moderate (+) and high (++) expression. C. Different degree of PI3KCA expression in OC. From left to right: negative (−), moderate (+) and high (++) expression. D. Different degree of PTEN expression in OC. From left to right: negative (−), reduced (−/+) and positive (+).Magnification 40X. Magnification of the insets 10X. (TIF) [file pone.0055362.s002.tif]

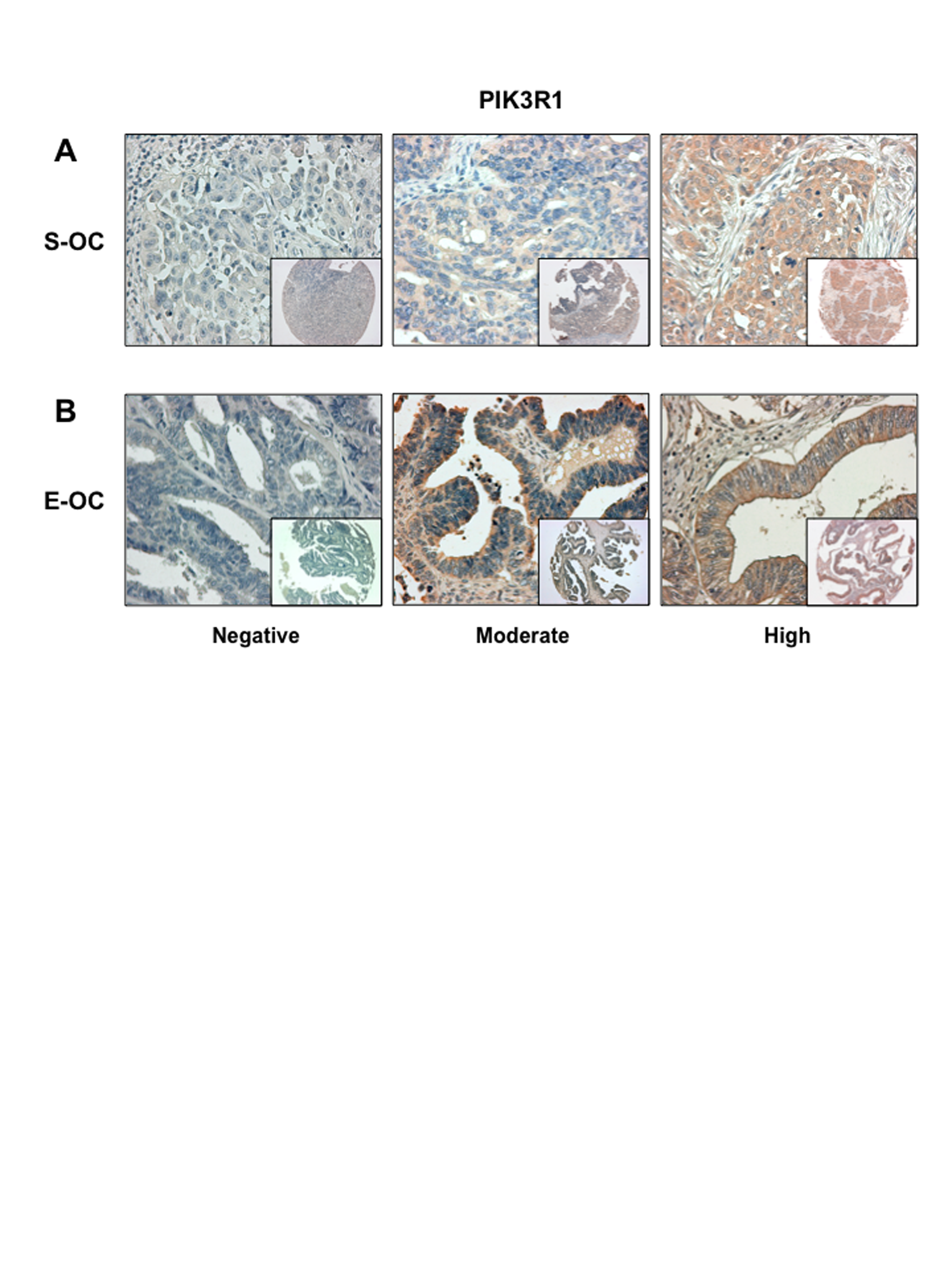

Supplement: Figure S3 — Immunostaining analysis of PIK3R1 in OC. A. Different degree of PIK3R1 expression in S-OC. From left to right: negative (−), moderate (+) and high (++) expression. B. Different degree of PIK3R1 expression in E-OC. From left to right: negative (−), moderate (+) and high (++) expression. Magnification 40X. Magnification of the insets 10X. (TIF) [file pone.0055362.s003.tif]

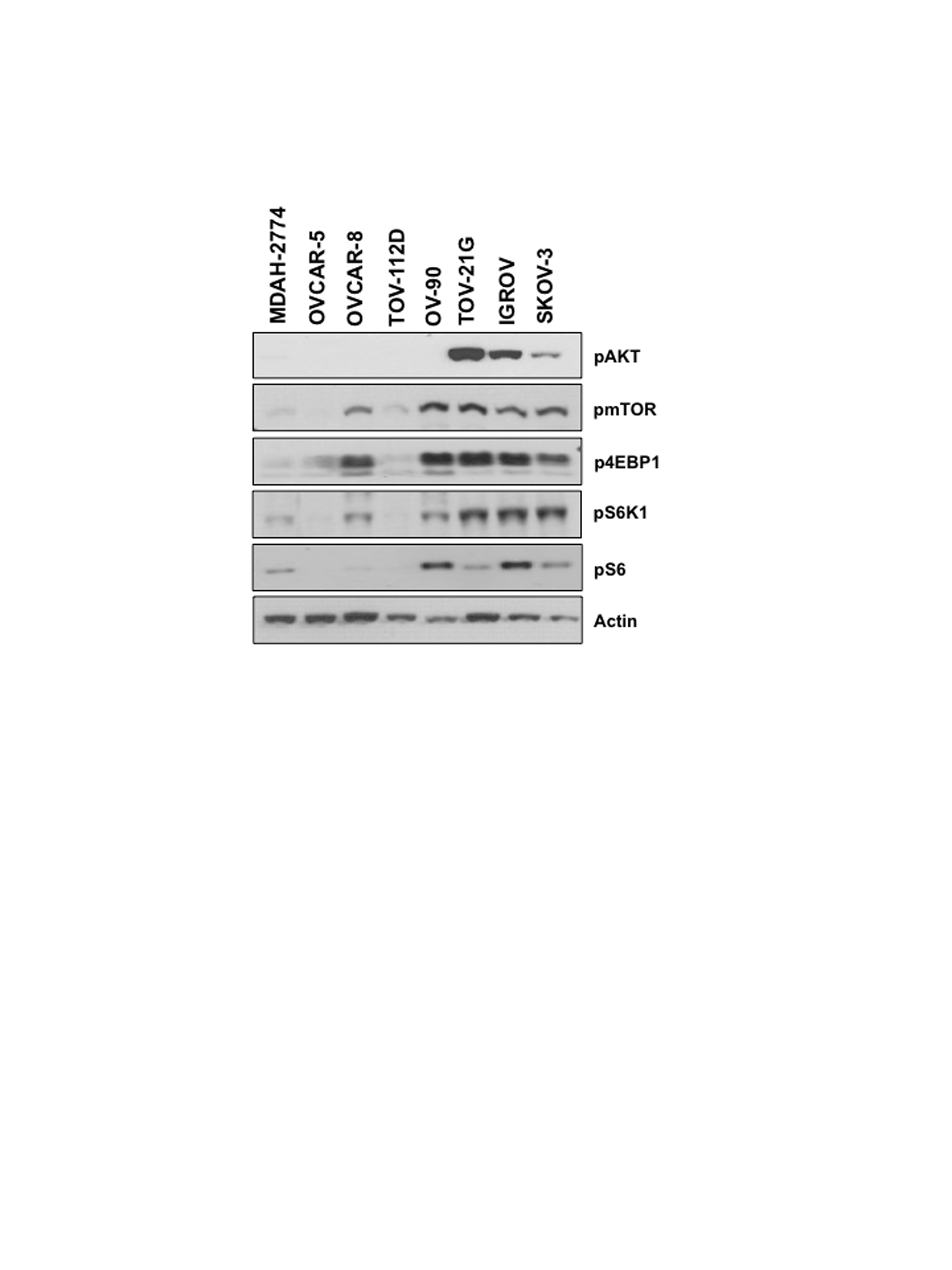

Supplement: Figure S4 — Analysis of pathways activated downstream PI3K in OC: mTOR and SGK3. Western blot analysis of phosphorylated AKT, mTOR, S6K1, S6 and 4EBP1 in ovarian cancer cell lines with absent (lanes 1–5) or present (lanes 6–8) genetic alterations that activate the PI3K/AKT pathway. (TIF) [file pone.0055362.s004.tif]
