# Supplementary material for: Multiple Genetic Alterations within the PI3K Pathway Are Responsible for AKT Activation in Patients with Ovarian Carcinoma
Source: PLoS One. 2013 Feb 7;8(2):e55362. doi: 10.1371/journal.pone.0055362 (PMC3567053; doi:10.1371/journal.pone.0055362)
Supplement: Table S1 — Samples distribution among hystotypes and clinico-pathological subclasses. (DOC) [file pone.0055362.s005.doc]

**Table S1. Samples distribution among hystotypes and clinico-pathological subclasses.**

|  | **S-OC** | **E-OC** | **CC-OC** | **Mu-OC** | **M-OC** | **TOTAL** |
| --- | --- | --- | --- | --- | --- | --- |
|  | **N=68** | **N=16** | **N=4** | **N=8** | **N=2** | **N=98** |
| **Age** |  |  |  |  |  |  |
| <58 | 33 | 8 | 1 | 6 | 1 | **49** |
| ≥58 | 35 | 8 | 3 | 2 | 1 | **49** |
| **Grade** |  |  |  |  |  |  |
| G1 | 1 | 3 | / | 3 | / | **7** |
| G2 | 3 | 9 | / | 5 | 1 | **18** |
| G3 | 64 | 4 | 4 | / | 1 | **73** |
| **FIGO** |  |  |  |  |  |  |
| I | 11 | 6 | 2 | 5 | / | **24** |
| II | 6 | 1 | 1 | 1 | / | **9** |
| III | 47 | 9 | 1 | 2 | 2 | **61** |
| IV | 4 | / | / | / | / | **4** |
| **N.I.^a^** |  |  |  |  |  |  |
| N0 | 26 | 9 | 3 | 4 | / | **42** |
| N1 | 26 | 5 | 1 | 1 | 2 | **35** |
| Nx | 16 | 2 | / | 3 | / | **21** |

**S-OC: S**erous **O**varian **C**arcinoma

**E-OC: E**ndometrioid **O**varian **C**arcinoma

**CC-OC: C**lear **C**ell **O**varian **C**arcinoma

**Mu-OC: M**ucinous **O**varian **C**arcinoma

**M-OC: M**ixed **O**varian **C**arcinoma

**^a^ N.I.:** Node Involvment.
