# Supplementary material for: Multiple Genetic Alterations within the PI3K Pathway Are Responsible for AKT Activation in Patients with Ovarian Carcinoma
Source: PLoS One. 2013 Feb 7;8(2):e55362. doi: 10.1371/journal.pone.0055362 (PMC3567053; doi:10.1371/journal.pone.0055362)
Supplement: Table S2 — Correlation between AKT activation and clinico-pathologic features of S-OC patients. (DOC) [file pone.0055362.s006.doc]

**Table S2. Correlation between AKT activation and clinico-pathologic features of S-OC patients.**

|  | **pAKT** | |  | |
| --- | --- | --- | --- | --- |
|  | **Negative** | **Positive** | | ***P value*** |
| **Age** |  |  | |  |
| <58 | 9 | 24 | | NS |
| ≥58 | 6 | 41 | |  |
| **Grade*^a^*** |  |  | |  |
| G1 | 0 | 1 | | NS |
| G2 | 1 | 2 | |  |
| G3 | 14 | 48 | |  |
| **FIGO stage *^a^*** |  |  | |  |
| **I** | 1 | 9 | | NS |
| **II** | 1 | 5 | |  |
| **III** | 12 | 34 | |  |
| **IV** | 1 | 3 | |  |

***^a^*** Limited to tumours for which both differentiation score/FIGO stage and pAKT staining were available (n= 66).

**NS**: not significant.
