# Supplementary material for: Multiple Genetic Alterations within the PI3K Pathway Are Responsible for AKT Activation in Patients with Ovarian Carcinoma
Source: PLoS One. 2013 Feb 7;8(2):e55362. doi: 10.1371/journal.pone.0055362 (PMC3567053; doi:10.1371/journal.pone.0055362)
Supplement: Table S4 — Patient-by-patient list of the genetic alterations observed in OC patients. (DOC) [file pone.0055362.s008.doc]

**TABLE S4. Patient-by-patient list of the genetic alterations observed in OC patients.**

| **Summary of genetic alterations in serous ovarian carcinoma** | | | | | | |
| --- | --- | --- | --- | --- | --- | --- |
| **Sample** | **Grade** | **FIGO** | **Copy number gain^a^** | **Mutation** | **PTEN^b^** | **pAKT^c^** |
| **S1** | G3 | IIIB | AKT1 (HP) |  | - | + |
| **S2** | G2 | IIIC | AKT1 (HP) |  | + | + |
| **S3** | G3 | IIIC |  |  | + | - |
| **S4** | G2 | IIIC | AKT2 (HP) PIK3CA (HP) |  | + | - |
| **S5** | G3 | IIIC | AKT1 (HP) AKT2 (HP) PIK3CA (HP) |  | - | + |
| **S6** | G3 | IIIC | PIK3CA (HP) |  | -/+ | + |
| **S7** | G3 | IB | AKT1 (HP) PIK3CA (HP) |  | + | - |
| **S8** | G3 | IIIB |  |  | + | - |
| **S9** | G3 | IIIC |  |  | ND | - |
| **S10** | G3 | IV | AKT1 (HP) |  | + | + |
| **S11** | G3 | IIIC |  |  | - | + |
| **S12** | G3 | IIIC |  |  | - | + |
| **S13** | G3 | IIIC |  |  | + | + |
| **S14** | G3 | IIIC |  |  | -/+ | + |
| **S15** | G3 | IIIC | AKT2 (HP) |  | -/+ | + |
| **S16** | G3 | IIIC |  |  | + | + |
| **S17** | G3 | IIIC |  |  | + | - |
| **S18** | G3 | IIIC |  |  | + | + |
| **S19** | G3 | IIIC | PIK3CA (HP) |  | + | + |
| **S20** | G1 | IC | AKT1 (A) PIK3CA (HP) |  | - | + |
| **S21** | G3 | IIIC | AKT2 (HP) |  | + | + |
| **S22** | G3 | IIIC |  |  | + | + |
| **S23** | G3 | IIIB | PIK3CA (HP) |  | + | - |
| **S24** | G3 | IB |  |  | + | + |
| **S25** | G2 | IIIC |  |  | + | + |
| **S26** | G3 | IIIC |  |  | + | + |
| **S27** | G3 | IIIC |  |  | ND | - |
| **S28** | G3 | IA |  |  | - | + |
| **S29** | G3 | IIIC |  |  | ND | ND |
| **S30** | G3 | IV | AKT1 (HP) PIK3CA (A) |  | -/+ | + |
| **S31** | G3 | IV | AKT1 (HP) |  | + | - |
| **S32** | G3 | IIIC |  |  | + | + |
| **S33** | G3 | IIIA |  |  | + | + |
| **S34** | G3 | IV |  |  | + | + |
| **S35** | G3 | IIIC |  |  | + | + |
| **S36** | G3 | IIIA |  |  | + | + |
| **S37** | G3 | IIA |  |  | - | + |
| **S38** | G3 | IIB |  |  | -/+ | + |
| **S39** | G3 | IIB |  |  | + | - |
| **S40** | G3 | IIIC |  |  | -/+ | + |
| **S41** | G3 | IC |  |  | + | + |
| **S42** | G3 | IIA |  |  | - | + |
| **S43** | G3 | IC |  |  | - | + |
| **S44** | G3 | IIIB | PIK3CA (A) |  | + | + |
| **S45** | G3 | IB |  |  | ND | ND |
| **S46** | G3 | IIIC | PIK3CA (A) |  | - | + |
| **S47** | G3 | IIIC |  |  | + | - |
| **S48** | G3 | IIIC |  |  | + | + |
| **S49** | G3 | IIIC | AKT1 A) |  | + | - |
| **S50** | G3 | IIIC | PIK3CA(A) |  | + | + |
| **S51** | G3 | IIA | AKT1 (A) AKT2 (A) PIK3CA (A) |  | + | + |
| **S52** | G3 | IA | AKT1 (A) AKT2 (HP) |  | - | + |
| **S53** | G3 | IIIC |  | KRAS G12V | + | + |
| **S54** | G3 | IIIB |  |  | -/+ | + |
| **S55** | G3 | IIIC | PIK3CA (A) |  | - | + |
| **S56** | G3 | IIIC | AKT2 (A) | PIK3CA E545A | + | + |
| **S57** | G3 | IIIB |  |  | ND | - |
| **S58** | G3 | IIIC | AKT2 (A) PIK3CA (A) |  | + | - |
| **S59** | G3 | IA |  |  | - | + |
| **S60** | G3 | IA | PIK3CA (A) |  | - | + |
| **S61** | G3 | IB |  | PIK3CA E545A | + | + |
| **S62** | G3 | IIIC | AKT1 (A) AKT2 (A) |  | + | + |
| **S63** | G3 | IIIC |  | PIK3CA E545A | + | - |
| **S64** | G3 | IIIB | PIK3CA (A) |  | + | + |
| **S65** | G3 | IIIA | PIK3CA (A) |  | + | + |
| **S66** | G3 | IIIC | PIK3CA (A) |  | + | + |
| **S67** | G3 | IIA | PIK3CA (A) |  | - | + |
| **S68** | G3 | IIIC |  |  | + | + |
| **Summary of genetic alterations in endometroid ovarian carcinoma** | | | | | | |
| **E1** | G2 | IC |  | PIK3CA E545A | + | + |
| **E2** | G2 | IA |  |  | ND | - |
| **E3** | G2 | IIIC | PIK3CA (HP) |  | - | + |
| **E4** | G1 | IA |  |  | ND | + |
| **E5** | G2 | IA | PIK3CA (HP) |  | + | + |
| **E6** | G2 | IIA | AKT1 (HP) |  | + | + |
| **E7** | G2 | IIIC |  |  | + | + |
| **E8** | G1 | IIIC |  |  | + | + |
| **E9** | G1 | IC |  |  | + | + |
| **E10** | G2 | IIIC |  |  | - | - |
| **E11** | G3 | IIIC |  |  | + | + |
| **E12** | G3 | IIIC |  |  | - | + |
| **E13** | G3 | IIIC |  | PIK3CA E545A | + | + |
| **E14** | G2 | IIIB | PIK3CA (A) |  | - | + |
| **E15** | G2 | IA |  |  | + | + |
| **E16** | G3 | IIIC |  |  | + | + |
| **Summary of genetic alterations in mucinous carcinoma** | | | | | | |
| **Mu1** | G2 | IC |  |  | ND | ND |
| **Mu2** | G2 | IIA |  |  | + | - |
| **Mu3** | G1 | IC |  |  | + | - |
| **Mu4** | G2 | IC | AKT1 (HP) |  | + | + |
| **Mu5** | G2 | IIIB |  |  | + | + |
| **Mu6** | G2 | IIIC |  | KRas G12R | - | + |
| **Mu7** | G1 | IA |  | KRas G13V | - | - |
| **Mu8** | G1 | IA |  |  | ND | ND |
| **Summary of genetic alterations in clear cells carcinoma** | | | | | | |
| **CC1** | G3 | IIA |  |  | + | + |
| **CC2** | G3 | IIIC |  |  | - | + |
| **CC3** | G3 | IA |  |  | - | + |
| **CC4** | G3 | IA | PIK3CA (A) |  | + | ND |
| **Summary of genetic alterations in mixed carcinoma** | | | | | | |
| **M1** | G2 | IIIC | AKT1(A) PIK3CA (A) |  | + | + |
| **M2** | G3 | IIIC | AKT1 (A) AKT2 (A) PIK3CA (A) |  | - | + |

**^a^** **LP**: Low Polisomy; **HP**: High Polisomy; **A**: Amplification

**^b^ +:** High; **+/-:** Reduced; **-:** Negative

**^c^ -:** Negative**; +:** Positive

**ND**: not determined.
