# Supplementary material for: Multiple Genetic Alterations within the PI3K Pathway Are Responsible for AKT Activation in Patients with Ovarian Carcinoma
Source: PLoS One. 2013 Feb 7;8(2):e55362. doi: 10.1371/journal.pone.0055362 (PMC3567053; doi:10.1371/journal.pone.0055362)
Supplement: Table S5 — Immunostaining evaluation of the members of the PI3K/AKT pathway. (DOC) [file pone.0055362.s009.doc]

**Table S5. Immunostaining evaluation of the members of the PI3K/AKT pathway.**

|  | **ND** | **Negative** | **%^a^** | **Moderate ^b^** | **%^a^** | **High** | **%^a^** | **Total** |
| --- | --- | --- | --- | --- | --- | --- | --- | --- |
| **AKT1** | 2 | 46 | **47.9** | 32 | **33.3** | 18 | **18.7** | 96 |
| **AKT2** | 10 | 36 | **40.9** | 41 | **46.6** | 11 | **12.5** | 88 |
| **PIK3CA** | 5 | 23 | **24.7** | 7 | **7.5** | 63 | **67.7** | 93 |
| **PIK3R1** | 4 | 12 | **12.8** | 13 | **13.8** | 69 | **73.4** | 94 |
| **PTEN** | 9 | 24 | **27** | 7 | **7.9** | 58 | **65.1** | 89 |

^a^ Referred to total number of samples analyzed.

^b^ Reduced as referred to reduced PTEN staining.
