# Supplementary material for: Multiple Genetic Alterations within the PI3K Pathway Are Responsible for AKT Activation in Patients with Ovarian Carcinoma
Source: PLoS One. 2013 Feb 7;8(2):e55362. doi: 10.1371/journal.pone.0055362 (PMC3567053; doi:10.1371/journal.pone.0055362)
Supplement: Table S6 — Immunostainng of the members of the PIK3/AKT pathway in different OC histotypes. (DOC) [file pone.0055362.s010.doc]

**Table S6. Immunostainng of the members of the PIK3/AKT pathway in different OC histotypes.**

|  | **ND (N)** | **Negative (N)** | **Moderate ^a^ (N)** | **High (N)** | **Total (N)** |
| --- | --- | --- | --- | --- | --- |
| **AKT1** |  |  |  |  |  |
| S-OC | 1 | 31 | 22 | 14 | 67 |
| E-OC | / | 6 | 7 | 3 | 16 |
| Mu-OC | 1 | 5 | 2 | / | 7 |
| CC-OC | / | 3 | 1 | / | 4 |
| M-OC | / | 1 | / | 1 | 2 |
| **AKT2** |  |  |  |  |  |
| S-OC | 7 | 23 | 31 | 7 | 61 |
| E-OC | / | 6 | 7 | 3 | 16 |
| Mu-OC | 3 | 4 | 1 | / | 5 |
| CC-OC | / | 3 | 1 | / | 4 |
| M-OC | / | / | 1 | 1 | 2 |
| **PIK3CA** |  |  |  |  |  |
| S-OC | 2 | 12 | 4 | 50 | 66 |
| E-OC | / | 6 | 2 | 8 | 16 |
| Mu-OC | 2 | 3 | / | 3 | 6 |
| CC-OC | 1 | 2 | / | 1 | 3 |
| M-OC | / | / | 1 | 1 | 2 |
| **PIK3R1** |  |  |  |  |  |
| S-OC | 2 | 7 | 8 | 51 | 66 |
| E-OC | / | 3 | 3 | 10 | 16 |
| Mu-OC | 2 | 2 | 2 | 2 | 6 |
| CC-OC | / | / | / | 4 | 4 |
| M-OC | / | / | / | 2 | 2 |
| **PTEN** |  |  |  |  |  |
| S-OC | 5 | 15 | 7 | 41 | 63 |
| E-OC | 2 | 4 | / | 10 | 14 |
| Mu-OC | 2 | 2 | / | 4 | 6 |
| CC-OC | / | 2 | / | 2 | 4 |
| M-OC | / | 1 | / | 1 | 2 |

^a^ Reduced PTEN staining.

**N**: number.
