# Supplementary material for: Multiple Genetic Alterations within the PI3K Pathway Are Responsible for AKT Activation in Patients with Ovarian Carcinoma
Source: PLoS One. 2013 Feb 7;8(2):e55362. doi: 10.1371/journal.pone.0055362 (PMC3567053; doi:10.1371/journal.pone.0055362)
Supplement: Table S7 — Correlation between AKT activation (pAKT) and the expression of the different members of the PI3K/AKT pathway in S-OC patients. (DOC) [file pone.0055362.s011.doc]

**Table S7. Correlation between AKT activation (pAKT) and the expression of the different members of the PI3K/AKT pathway in S-OC patients.**

|  |  | **pAKT** | |  |  |
| --- | --- | --- | --- | --- | --- |
|  |  | **Negative** | **Positive** | **N°** | **P value** |
| **AKT1^a^** | **Negative** | 12 | 18 | 30 | 0.009 |
|  | **Moderate** | 2 | 20 | 22 |  |
|  | **High** | 1 | 13 | 14 |  |
| **AKT2^a^** | **Negative** | 7 | 15 | 22 | NS |
|  | **Moderate** | 4 | 27 | 31 |  |
|  | **High** | 0 | 7 | 7 |  |
| **PIK3CA^a^** | **Negative** | 8 | 4 | 12 | <0.0001 |
|  | **Moderate** | 0 | 4 | 4 |  |
|  | **High** | 7 | 43 | 50 |  |
| **PIK3R1^a^** | **Negative** | 4 | 3 | 7 | <0.0001 |
|  | **Moderate** | 5 | 3 | 8 |  |
|  | **High** | 6 | 45 | 51 |  |
| **PTEN^a^** | **Positive** | 12 | 29 | 41 | 0.019 |
|  | **Reduced** | 0 | 7 | 7 |  |
|  | **Negative** | 0 | 15 | 15 |  |

***^a^*** Patients for which pAKT staining was available (N°).

**NS**: not significant.
