# Supplementary material for: Multiple Genetic Alterations within the PI3K Pathway Are Responsible for AKT Activation in Patients with Ovarian Carcinoma
Source: PLoS One. 2013 Feb 7;8(2):e55362. doi: 10.1371/journal.pone.0055362 (PMC3567053; doi:10.1371/journal.pone.0055362)
Supplement: Table S8 — Correlation between AKT activation (pAKT) and the expression of the different members of the PI3K/AKT pathway in E-OC patients. (DOC) [file pone.0055362.s012.doc]

**Table S8. Correlation between AKT activation (pAKT) and the expression of the different members of the PI3K/AKT pathway in E-OC patients.**

|  |  | **pAKT** | |  |  |
| --- | --- | --- | --- | --- | --- |
|  |  | **Negative** | **Positive** | **Total** | **P *value*** |
| **AKT1^a^** | **Negative** | 2 | 4 | 6 | NS |
|  | **Moderate** | 0 | 7 | 7 |  |
|  | **High** | 0 | 3 | 3 |  |
| **AKT2^a^** | **Negative** | 2 | 4 | 6 | NS |
|  | **Moderate** | 0 | 7 | 7 |  |
|  | **High** | 0 | 3 | 3 |  |
| **PIK3CA*^a^*** | **Negative** | 2 | 4 | 6 | NS |
|  | **Moderate** | 0 | 2 | 2 |  |
|  | **High** | 0 | 8 | 8 |  |
| **PIK3R1*^a^*** | **Negative** | 1 | 2 | 3 | NS |
|  | **Moderate** | 0 | 3 | 3 |  |
|  | **High** | 1 | 9 | 10 |  |
| **PTEN*^a^*** | **Positive** | 0 | 10 | 10 | NS |
|  | **Reduced** | 0 | 0 | 0 |  |
|  | **Negative** | 1 | 3 | 4 |  |

**^a^** Patients for which pAKT staining was available (N°).

**NS**: not significant.
