# Supplementary material for: Multiple Genetic Alterations within the PI3K Pathway Are Responsible for AKT Activation in Patients with Ovarian Carcinoma
Source: PLoS One. 2013 Feb 7;8(2):e55362. doi: 10.1371/journal.pone.0055362 (PMC3567053; doi:10.1371/journal.pone.0055362)
Supplement: Table S9 — Correlation between alterations in the expression of PTEN, PIK3CA, AKT1 and AKT2 and pAKT status in S-OC. (DOC) [file pone.0055362.s013.doc]

**Table S9. Correlation between alterations in the expression of PTEN, PIK3CA, AKT1 and AKT2 and pAKT status in S-OC.**

| **Alteration** | **pAKT negative (N=13)** | **pAKT positive (N=50)** |
| --- | --- | --- |
| AKT1 ***^a^*** | 1 | 1 |
| AKT2 ***^b^*** | 0 | 0 |
| PIK3CA ***^c^*** | 7 | 20 |
| PTEN ***^d^*** | 0 | 1 |
| AKT1, PTEN | 0 | 1 |
| AKT2, PTEN | 0 | 0 |
| PIK3CA, PTEN | 0 | 10 |
| AKT1, AKT2 | 0 | 1 |
| PIK3CA, AKT1 | 0 | 5 |
| PIK3CA, AKT2 | 0 | 4 |
| AKT1, AKT2, PTEN | 0 | 0 |
| AKT1, PIK3CA, PTEN | 0 | 3 |
| AKT2, PIK3CA, PTEN | 0 | 0 |
| AKT1, AKT2, PIK3CA | 0 | 2 |
| AKT1, AKT2, PIK3CA, PTEN | 0 | 0 |

**^a^** High AKT1 expression as defined in the manuscript.

**^b^** High AKT2 expression as defined in the manuscript.

**^c^** High PIK3CA expression as defined in the manuscript.

**^d^** PTEN loss as defined in the manuscript.
