# Supplementary material for: Multiple Genetic Alterations within the PI3K Pathway Are Responsible for AKT Activation in Patients with Ovarian Carcinoma
Source: PLoS One. 2013 Feb 7;8(2):e55362. doi: 10.1371/journal.pone.0055362 (PMC3567053; doi:10.1371/journal.pone.0055362)
Supplement: Materials and Methods S1 — PIK3CA mutational analysis (Light cycler). Cell lines and treatment. Primer sequences. (DOC) [file pone.0055362.s015.doc]

**Supporting Materials and Methods**

**PIK3CA mutation analysis (LightCycler)**

For exon 9 genotyping, the following sensor (LC Red 640 AGGATCTCGTGTAGAAATTGCTTTGAGCTGTTCTT-phoshate) and anchor (TTTCTCCTGCTCAGTGATTTCAGAGA-Fluorescein) probes were used. The cycling conditions were as follows: initial denaturation at 95 °C for 10 min, followed by 45 cycles at 95 °C for 10 s, 58 °C for 15 s, and 72 °C for 8 s. For exon 20 genotyping, the following sensor (ACCCTAGCCTTAGATAAAACTGAGCAAGAGGCTTT-Fluorescein) and anchor (LC Red 640 GAGTATTTCATGAAACAAATGAATGCACATC) probes were used. The cycling conditions were as follows: initial denaturation at 95 °C for 10 min, followed by 45 cycles at 95 °C for 10 s, 59 °C for 15 s, and 72 °C for 7 s.

**Cell lines and treatment**

The human ovarian cancer cell lines used in this work were provided by dr. Gustavo Baldassarre (CRO, Aviano, Italy). MDAH-2774, SKOV3, IGROV, OVCAR-5, OVCAR-8 and OVCAR-429 were maintained in RPMI 1640 supplemented with 10% foetal bovine serum; OV90, TOV112D, TOV21G in MCDB105 were maintained in M199 medium supplemented with 15% foetal bovine serum (Sigma-Aldrich). LY294002 and RAD001 were from Sigma Aldrich. Cells were treated for 24h with 20μM LY294002, 20nM RAD001 or combinations thereof.

**Primer sequences**

| **PRIMER ID** | **SEQUENCE** |
| --- | --- |
| **SEQUENCE ANALYSIS** |  |
| PIK3CA EX9 FW | 5’-CAGAGTAACAGACTAGCTAG -3’ |
| PIK3CA EX9 REV | 5’-TAGCACTTACCTGTGACTCC -3’ |
| KRAS EX2 FW | 5’-GACTGAATATAAACTTGTGG-3’ |
| KRAS EX2 REV | 5’-CTGTATCAAAGAATGGTCCT-3’ |
| KRAS EX3 FW | 5’-TTTTTGAAGTAAAAGGTGCACTGTA-3’ |
| KRAS EX3 REV | 5’-ATATTATATGCATGGCATTAGCAAAG-3’ |
| **Q-PCR** |  |
| PTEN FW | 5’-GTTTGATTGCTGCATATTTCAG-3’ |
| PTEN REV | 5’ CCTGTATACGCCTTCAAGTC-3’ |
| PIK3R1 FW | 5’- atggctcctgcactcttcat-3’ |
| PIK3R1 REV | 5’- CAGGTTTTCAGCTTTGTTTCG-3’ |
| GAPDH FW | 5’-GGGCTGCTCACATATTCTGGA-3’ |
| GAPDH REV | 5’-CGCCCAATACGACCAAATCT-3’ |
| **Q-RT-PCR** |  |
| PTEN FW  PTEN REV | 5’-AATCCTCAGTTTGTGGTCT-3’  5’ GGTAACGGCTGAGGGAACT-3’ |
| PIK3R1 FW | 5’- GCTGAATGGTACTGGGGAGA-3’ |
| PIK3R1 REV | 5’-TACCAAAAAGGTCCCGTCTG-3’ |
| HMGA-1 FW | 5’- GAAGGAGCCCAGCGAAGTG-3’ |
| HMGA-1 REV | 5’-TTCTCCAGTTTTTTGGGTCTGC-3’ |
| JUN-B FW | 5’-ACTCATACACAGCTACGGGATACG-3’ |
| JUN-B REV | 5’-GGCTCGGTTTCAGGAGTTTG-3’ |
| FOS FW | 5’-CCGGGGATAGCCTCTCTTAC-3’ |
| FOS REV | 5’-GTGGGAATGAAGTTGGCACT-3’ |
| MYC FW | 5′-TCAAGAGGCGAACACACAAC-3′ |
| MYC REV | 5′-GGCCTTTTCATTGTTTTCCA-3′ |
| GAPDH FW | 5’-GAGTCAACGGATTTGGTCGT-3’ |
| GAPDH REV | 5’-GACAAGCTTCCCGTTCTCAG-3’ |
